# Parasitic behavior in competing chemically fueled reaction cycles†

**DOI:** 10.1039/d1sc01106e

**Published:** 2021-04-28

**Authors:** Patrick S. Schwarz, Sudarshana Laha, Jacqueline Janssen, Tabea Huss, Job Boekhoven, Christoph A. Weber

**Affiliations:** Department of Chemistry, Technical University of Munich Lichtenbergstraße 4 85748 Garching Germany job.boekhoven@tum.de; Biological Physics, Max Planck Institute for the Physics of Complex Systems Nöthnitzer Straße 38 01187 Dresden Germany weber@pks.mpg.de; Center for Systems Biology Dresden Pfotenhauerstraße 108 01307 Dresden Germany; Institute for Advanced Study, Technical University of Munich Lichtenbergstraße 2a 85748 Garching Germany

## Abstract

Non-equilibrium, fuel-driven reaction cycles serve as model systems of the intricate reaction networks of life. Rich and dynamic behavior is observed when reaction cycles regulate assembly processes, such as phase separation. However, it remains unclear how the interplay between multiple reaction cycles affects the success of emergent assemblies. To tackle this question, we created a library of molecules that compete for a common fuel that transiently activates products. Often, the competition for fuel implies that a competitor decreases the lifetime of these products. However, in cases where the transient competitor product can phase-separate, such a competitor can increase the survival time of one product. Moreover, in the presence of oscillatory fueling, the same mechanism reduces variations in the product concentration while the concentration variations of the competitor product are enhanced. Like a parasite, the product benefits from the protection of the host against deactivation and increases its robustness against fuel variations at the expense of the robustness of the host. Such a parasitic behavior in multiple fuel-driven reaction cycles represents a lifelike trait, paving the way for the bottom-up design of synthetic life.

## Abstract 2

Non-equilibrium, fuel-driven reaction cycles serve as model systems of the intricate reaction networks of life.

## Graphical abstract

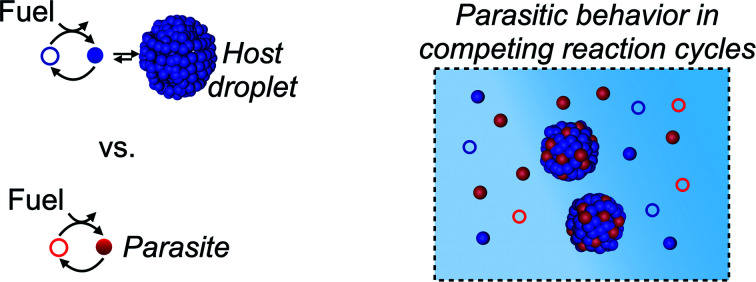

## Introduction

In chemically fueled systems, the propensity of molecules to form assemblies is regulated by a chemical reaction cycle. In the cycle, a precursor is activated at the expense of a chemical fuel.^1–8^ Simultaneously, a deactivation reaction spontaneously reverts the product to its precursor state. Thus, a population of transient product molecules emerges whose properties are regulated kinetically. In recent years, examples of such reaction cycles have been introduced that regulate the ability of molecules to assemble or phase-separate, resulting in dynamic structures like colloids,^9,10^ fibers,^10–16^ supramolecular polymers,^17–19^ oil-based droplets,^20,21^ coacervate-based droplets,^22,23^ vesicles,^24–26^ micelles,^27^ particle clusters,^28–31^ macrocycles^32^ and DNA-based nanostructures.^33,34^ Due to the transient nature of these building blocks, these assemblies are endowed with properties typically absent at thermodynamic equilibrium. For example, fibrils that spontaneously self-divide,^23^ temporary hydrogels,^10,35–37^ or oil-based emulsions of which ripening is accelerated.^2^ Moreover, the theory on active emulsions suggests that droplets can self-divide.^38,39^ More recently, examples of assemblies were observed that exert feedback over their chemical reaction cycle.^11,27,40,41^ The underlying mechanisms can result in exciting behavior like the spontaneous emergence of switches between the morphologies or the ability of molecules to persist while others decay.^9,20^ All these developments in the field are incremental steps towards the synthesis of life, and a living system essentially represents a complex non-equilibrium assembly of molecules that is regulated by chemical reaction cycles.^42–44^ However, in living systems, a vast number of reaction cycles operate simultaneously and interact in intricate networks through feedback mechanisms. While such systems show interesting and complex emergent properties in a close-to-equilibrium context,^45–50^ the behavior of multiple reaction cycles in fuel-driven synthetic systems has been underexplored. In particular, competition and feedback are expected to lead to interesting emergent behavior in such systems. For example, oil droplets showed that selection and inhibition can occur in systems competing for a common fuel.^51^

In this work, we show an unexpected behavior in phase-separated emulsions that are regulated by chemical reaction cycles and compete for a fuel. Counterintuitively, the lifetime of a transient product can be vastly prolonged even when resources have to be shared. The underlying mechanism is based on co-phase separation which protects products against deactivation, and it shows similarity to how a parasite benefits from the presence of a host.

## Results and discussion

**Fig. 1:**
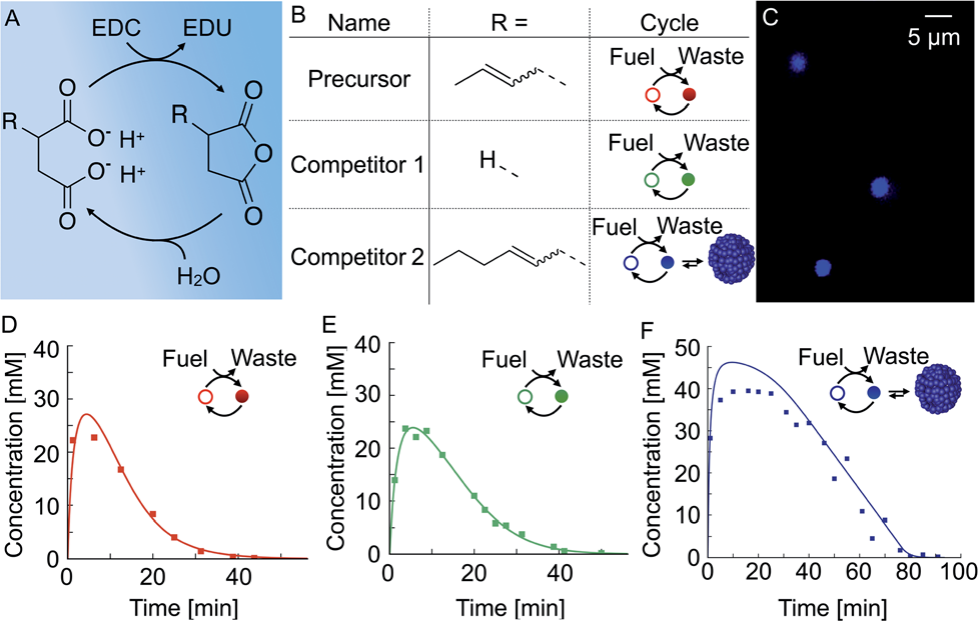
The design of chemically fueled reaction cycles. (A) The chemically fueled reaction cycle used in this work. Succinate derivatives are converted into their corresponding transient anhydrides. (B) Molecular structures of the precursor, competitor 1, and competitor 2. The cycle column shows a schematic representation of the cycle with the succinate derivative as an open circle and the anhydride as a closed circle. Competitor 2 can form droplets. (C) Confocal microscopy of 50 mM competitor 2 fueled with 100 mM EDC. The corresponding anhydride product phase-separates into micron-sized oil droplets. (D–F) The anhydride product concentration profile of 50 mM precursor (D), competitor 1 (E), and competitor 2 (F) fueled with 100 mM EDC. Markers represent HPLC data; solid lines represent data calculated using the theoretical kinetic model.

We used a chemical reaction cycle that is driven by the hydration of the condensing agent EDC (fuel, 1-ethyl-3-(3-di-methylaminopropyl)carbodiimide). In the activation reaction, the fuel condenses a succinate derivative into its corresponding anhydride product (Fig. 1A). In the aqueous media, the corresponding anhydride rapidly hydrolyses to the initial succinate derivative. We refer to this reaction step as the deactivation. Similar to the fuel-driven reaction cycles in biological systems, the energy obtained from the hydrolysis of EDC is used for the transient activation of the succinate derivative. The population of anhydride product can thus only be maintained when the rate of activation equals the rate of deactivation.^52^

In this study, we used three succinate derivatives: 2-buten-1-ylsuccinate, which we refer to as precursor, succinate (competitor 1) and 2-hexen-1-ylsuccinate (competitor 2, Fig. 1B). We observed that the addition of fuel to competitor 2 made the solution turn turbid due to the presence of oil-droplets which we verified *via* confocal microscopy (Fig. 1C), and is in line with previous work.^53^ However, the emergence of droplets could not be observed for the precursor and competitor 1.

In order to determine the kinetics of the three reaction cycles, we fueled 50 mM of each succinate derivative with 100 mM EDC and quantified the corresponding anhydride product concentration by means of high-performance liquid chromatography (HPLC). When we fueled 50 mM precursor with 100 mM EDC, we found that the precursor is immediately converted to roughly 25 mM of the product and, after the depletion of the fuel, degraded rapidly with a first-order decay within 24 minutes (Fig. 1D). Next, we fueled 50 mM of competitor 1 with 100 mM EDC and observed a similar yield and lifetime (Fig. 1E). In contrast, under the same conditions, fueling competitor 2 resulted in 45 mM anhydride product which lasted for over an hour (Fig. 1F). We explain the increased yield and lifetime of the droplet-forming anhydride product of competitor 2 by a previously described self-protection mechanism, *i.e.*, the phase separated anhydride product is shielded from water and thus protected from hydrolysis.^9,20,53^ Consequently, hydrolysis occurs only on the anhydride molecules in solution which we refer to as the outside equilibrium concentration of the anhydride product (*c*_out_). The hydrolysis rate can then be calculated by *r* = *k*_d_*c*_out_, where *k*_d_ is the hydrolysis rate constant. Since both *k*_d_ and *c*_out_ are constant, the effective hydrolysis rate is constant leading to a linear decay of the total anhydride product concentration when all fuel is consumed. Indeed, when using this equation in a theoretical kinetic model, we can accurately predict the concentration of fuel, succinate derivative and anhydride product for all three chemical reaction cycles (solid lines in Fig. 1D–F).

**Fig. 2:**
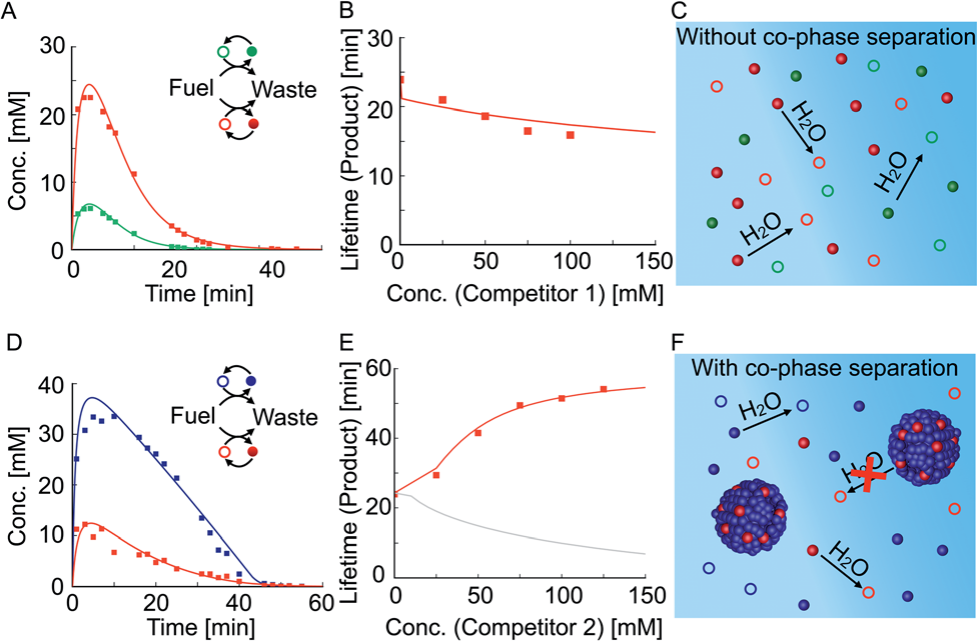
Competition between reaction cycles for a shared fuel. (A) The anhydride concentration profiles when 50 mM precursor (red) and 50 mM competitor 1 (green) compete for 100 mM fuel. (B) The lifetime of the product against the concentration of competitor 1. The lifetime decreases with increasing competition. (C) Schematic representation of the hydrolysis of anhydrides in the experiment in (A). (D) The anhydride concentration profiles when 50 mM precursor (red) and 50 mM competitor 2 (blue) compete for 100 mM fuel. (E) The lifetime of the product against the concentration of competitor 2 (red). Using the theoretical kinetic model, we show that for the same system, but in the absence of co-phase separation, the lifetime decreases (gray line). (F) Schematic representation of the hydrolysis of anhydrides in the presence of droplets. Markers represent HPLC data; solid lines represent data calculated using the theoretical kinetic model.

We tested how the kinetics of the reaction cycles are affected when the precursor competes with either competitor 1 or competitor 2 for fuel. We were particularly interested in how the anhydride products influence each other's activation and deactivation reactions and thereby determine their lifetimes. When we mixed equal concentrations of the precursor with competitor 1 and fueled with 100 mM EDC, we found lower yields and shorter reaction cycles for each of the anhydrides compared to their respective non-competing reaction cycles (Fig. 2A*versus*Fig. 1D and E). In order to quantify this effect, we measured the lifetime of the product as a function of competitor 1 concentration, keeping the precursor concentration fixed at 50 mM (Fig. 2B). The lifetime is defined as the time period during which the average product concentration exceeds a chosen threshold of 2 mM (see ESI Section 4† for a discussion on the robustness of the results for different threshold values). Briefly, the threshold concentration of 2 mM was chosen as it is equal to the *c*_out_ of competitor 2 which means that droplets dissolve below this threshold. Moreover, the threshold value is not in the tailing regime of the exponential decay of the anhydrides allowing to capture the effects of phase separation on product lifetime (ESI Fig. 11†). We find that the lifetime decreases with increasing the concentration of competitor 1, given the fact that the precursor and competitor 1 now have less fuel at their disposal compared to their corresponding non-competing reaction cycles (ESI Fig. 7†). The anhydrides of both reaction cycles are present side by side and hydrolyze in the aqueous media (Fig. 2C). In summary, both reaction cycles suffer from the competition for fuel.

The relation between the lifetime and amount of competitor was very different when the precursor competed with competitor 2, which can phase-separate. Despite the competition for fuel, the lifetime of the product increased with increasing competitor 2 concentration (Fig. 2D and E). When 50 mM of competitor 2 was added, the lifetime of the product increased to 43 minutes and the decay suddenly differed from the previously observed first-order decay (Fig. 2D). The increased lifetime is particularly surprising considering that the maximum yield of the product decreased from roughly 25 mM to 10 mM when competitor 2 was added (Fig. 1D*versus*Fig. 2D). In contrast, the lifetime of the product of competitor 2 decreased from 77 minutes when on its own to 43 minutes when competing with the precursor for fuel (Fig. 1F*versus*Fig. 2D). Moreover, we found that the maximum yield of the product of competitor 2 decreased from roughly 45 mM to 35 mM when competing with the precursor for fuel. In summary, the product of competitor 2 suffers whilst the product benefits from the competition for fuel between the reaction cycles. Interestingly, both anhydrides had the same lifetime indicating a coupling between the two reaction cycles. When we further increased the concentration of competitor 2 while fixing the precursor concentration, thelifetime of the product increased even further (Fig. 2E and ESI Fig. 9†). We hypothesize that the counterintuitive behavior is related to the ability of the product to co-phase separate with the product of competitor 2. Thus, the product benefits from the self-protection mechanism of the droplets formed by the product of competitor 2 (Fig. 2F). In other words, co-phase separation decreases the concentration of the product in the aqueous phase and thereby its deactivation rate.

We investigated the composition of the oil phase during the reaction cycle by centrifugation and HPLC. We found that the product is indeed part of the oil phase (ESI Fig. 13A–C†). Moreover, when we increased the concentration of competitor 2, we found that the composition of the oil phase changed, which suggests that the composition of the oil phase is dictated by the two reaction cycles. We also measured the composition of the aqueous phase after 16 minutes in the reaction for various competitor 2 concentrations (ESI Fig. 2A and B†). Assuming that the system is close to local phase separation equilibrium (see ESI Section 2† for an estimate supporting this assumption), the concentrations of the anhydrides in the aqueous phase are approximately equal to their outside equilibrium concentration *c*_out_. We found an almost constant *c*_out_ of roughly 2 mM for the anhydride product of competitor 2 in the presence or absence of the precursor (ESI Fig. 2A†). In other words, the *c*_out_ of the product of competitor 2 was hardly affected by the presence of the product. In contrast, we found that the *c*_out_ of the product decreased drastically, ranging from roughly 28 mM without competition to 0.6 mM with 125 mM concentration of competitor 2 (ESI Fig. 2B†).

**Fig. 3:**
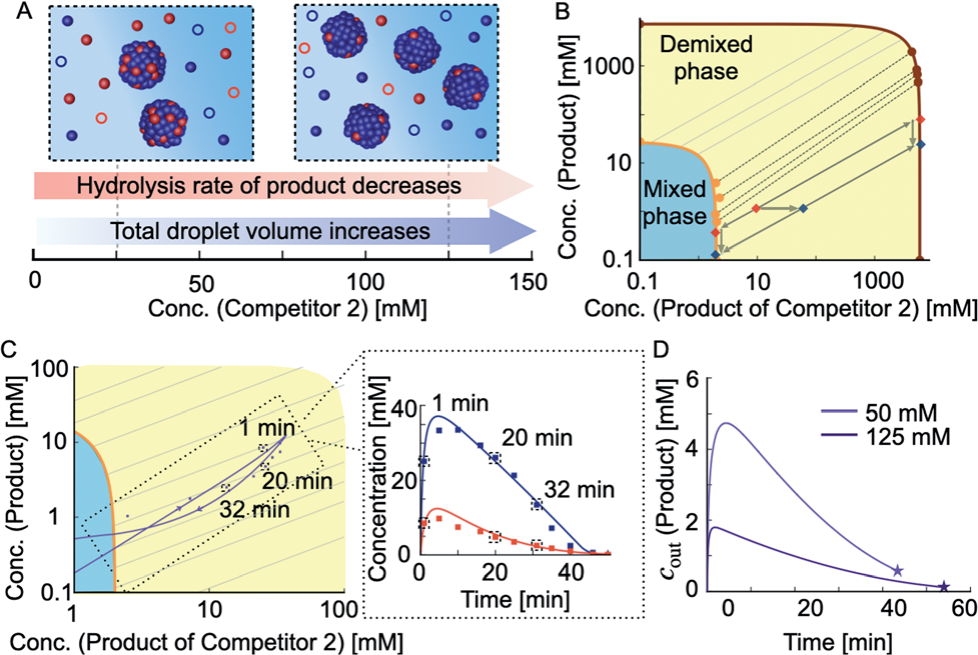
Mechanism of co-phase separation and increased lifetime. (A) Schematic representation of how increasing concentration of competitor 2 affects co-phase separation and thereby the hydrolysis rate of the product. (B) Ternary phase diagram depicting the equilibrium concentrations of the product and the product of competitor 2. The circles correspond to the experimental data and the solid lines represent the theoretical binodal and tie lines. The dashed black lines represent the experimental tie lines. (C) The trace of total concentrations of product and product of competitor 2 in the phase diagram during the reaction cycle when 50 mM precursor and 50 mM competitor 2 compete for 100 mM fuel. The arrows depict the direction in which the total concentrations move with time. Markers represent HPLC data; solid lines represent data calculated using the theoretical kinetic model. (D) The outside equilibrium concentration *c*_out_ of the product over time for 50 mM and 125 mM competitor 2. The course of the *c*_out_ of the product is dictated by the shape of the orbital in the phase diagram and the tie lines it crosses. The stars denote the point of dissolution of the droplets.

The results described above suggest that co-phase separation takes place and that co-phase separation protects both anhydride products from hydrolysis. Thus, the product of competitor 2 serves as a host for the product and protects it from hydrolysis-driven deactivation. We assumed that competition affects the co-phase separation as it results in an increased total droplet volume and a decreased hydrolysis rate of the product (Fig. 3A). To understand the full implications of this relation, we derived a model that accounts for the interplay between the chemical reaction kinetics and the physics of phase separation. The latter is determined by the phase diagram of the co-phase separating anhydride components (Fig. 3B and C). Since diffusion is fast compared to the hydrolysis of both anhydrides, changes in their total concentrations due to chemical reactions are slow enough such that phase separation can equilibrate quasi-instantaneously (see ESI Section 2†). Thus, the non-equilibrium chemical kinetics changes the average product concentrations leading to an orbit in the equilibrium phase diagram.

To determine the phase diagram in the experimental system, we measured the total anhydride concentrations at 16 minutes into the cycle and subtracted the previously determined concentrations in the aqueous phase to find the amount of each anhydride in the oil droplets (Fig. 3B and ESI Fig. 2A–C, see ESI Tables 4 and 5† and Methods). Together with the molecular volumes, we can thus calculate the concentrations of both anhydrides in the oil phase. In other words, for each set of total anhydride concentrations in the system corresponding to different initial competitor 2 concentrations, we calculated the anhydride concentrations in the aqueous phase and the oil phase (Fig. 3B, markers on the orange and dark red line, respectively). The measured concentrations which correspond to the coexisting phases in the phase diagram can be connected by tie lines (Fig. 3B, dashed lines between the orange and dark red line). We fitted the ternary Flory–Huggins model to the experimentally determined values.^54^ We found good agreement between the experimentally measured tie lines and the theoretically calculated ones (Fig. 3B). The theoretical phase diagram also interpolated between the experimentally measured data points. With this interpolation, we could determine the anhydride concentrations inside and outside of the droplets (oil phase) for any total concentration of anhydrides. As an example, when the total concentrations in the system were measured to be 1 mM product and 10 mM product of competitor 2, the tie line connects to concentrations of the anhydrides in the aqueous phase of 0.5 mM of product and 2 mM of the product of competitor 2 (Fig. 3B, red marker). In other words, under these conditions, roughly half of the product was protected. The phase diagram also showed that if the total concentration of product of competitor 2 increased (total concentration of product remaining constant at 1 mM), the system shifted to another tie line, and the *c*_out_ of the product decreased further (Fig. 3B, blue marker).

In the following, we extend the previously described kinetic model for two competing reaction cycles^53^ and account for the physics of co-phase separation characterized by the phase diagram. The kinetic model determines the time-dependent concentrations of fuel, succinate derivatives and anhydride products at each second of the reaction cycle *via* a set of five differential equations. The extended kinetic model in addition takes into account the concentrations in the aqueous phase and the oil phase and considers that activation and deactivation only take place in the aqueous phase. Solving the underlying kinetic equations of the extended model, we found that the calculated data was in good agreement with the concentrations measured by HPLC (Fig. 2D and ESI Fig. 2†). The model also allowed to represent the theoretical data and the HPLC data as points along orbits in the phase diagram (Fig. 3C). Each data point on such an orbit can be decomposed into concentrations of the anhydrides in the aqueous and in the oil phase. If an orbit lies parallel to a tie line, the anhydride concentrations in the aqueous phase remain almost constant over time. This implies that both anhydrides hydrolyze *via* kinetics close to zeroth-order as long as droplets are present. However, if the orbit evolves through several tie lines, the product concentration in the aqueous phase changes with time. In other words, the *c*_out_ of the product of competitor 2 in the aqueous phase barely changes and is independent of the shape of the orbit, *i.e.*, hydrolysis occurs *via* zeroth-order kinetics with or without the product.

In contrast, the *c*_out_ of the product changed drastically with the amount of competitor 2, and its time-dependent evolution depends on the shape of the orbit through the phase diagram. The extended kinetic model allowed us to calculate the outside equilibrium concentration *c*_out_ of the product as a function of time for different competitor 2 concentrations (Fig. 3D). For low concentration of competitor 2, the *c*_out_ of the product varied drastically from roughly 5 mM to 0.7 mM over the course of the reaction cycle. In contrast, for high concentration of competitor 2, the *c*_out_ varied only from roughly 2 mM to 0.5 mM. In summary, we showed that the shape of the orbit is influenced by the amount of competitor 2, *i.e.*, the more competitor is present, the more parallel the orbits are oriented with respect to the tie lines (ESI Fig. 14A–F†). However, due to adding fuel only at the beginning of the kinetics, all systems show a single orbit that enters and leaves the domain of co-phase separation in the phase diagram.

**Fig. 4:**
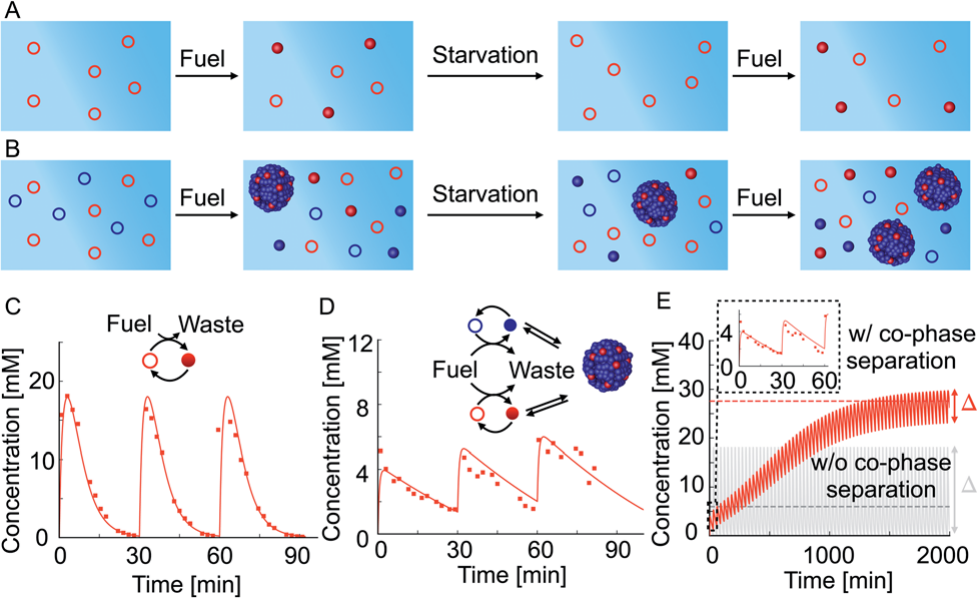
Co-phase separation facilitates the survival of the product in repetitive fueling starvation experiments. (A and B) Schematic representation of the precursor in periodic fueling and starvation periods without (A) and with (B) competitor 2. (C) 50 mM precursor fueled with an amplitude of 60 mM every 30 minutes. (D) 50 mM precursor and 100 mM competitor 2 fueled with an amplitude of 60 mM every 30 minutes. Markers represent HPLC data; solid lines represent data calculated using the theoretical kinetic model. (E) Calculation using the theoretical kinetic model of long-time kinetics of the reaction cycle of the experiments in (C) and (D). The gray and red dashed lines represent the mean concentrations achieved at pseudo steady state without and with co-phase separation, respectively. Inset shows the first three refueling steps. Note that the oscillations around the pseudo steady state concentration are severely damped with co-phase separation.

We tested how co-phase separation is affected when the system is subject to periodic fueling and starvation periods. We chose the amount of fuel and fueling frequency such that the product is depleted during each starvation period (Fig. 4A). We hypothesize that competition with competitor 2 under the exact same conditions let the product survive starvation (Fig. 4B). Indeed, when we periodically fueled 50 mM precursor every 30 minutes with 60 mM of fuel, we found that the corresponding product completely hydrolyzed after each starvation period (Fig. 4C). In contrast, when we periodically fueled 50 mM precursor and 100 mM competitor 2 with the same amplitude and frequency, we observed that co-phase separation protected the product from hydrolysis and thereby helped it to survive starvation (Fig. 4D and ESI Fig. 15A†). Despite the competition and lower anhydride yield, the survival of the product during the starvation period resulted in a drastically increased yield over fueling and starvation periods compared to a system without competitor 2 which did not show an increased yield. We used our theoretical kinetic model to calculate the response of the system to hundreds of cycles (Fig. 4E and ESI Fig. 15B–D†). We found that co-phase separation of the product with the anhydride product of competitor 2 resulted in a pseudo-steady state of the product in which the concentration oscillated around roughly 27 mM (red dashed line in Fig. 4E). In contrast, in the absence of competitor 2, the product oscillated around a mean concentration of roughly 6 mM and did not show any increase in concentration over time (gray solid line in Fig. 4E). These observations support the idea that the product of competitor 2 acts as a host and that the product of the precursor thus survives longer, benefitting like a parasite.

Besides the anticipated result of survival in the presence of a host, we found a surprising new behavior, *i.e.*, we observed that the oscillation in the concentrations in pseudo steady state due to fueling and starvation was dependent on the amount of competitor in the system (Fig. 4E). Specifically, in the first experiment, the concentration oscillated between a maximum of 18 mM and a minimum of 0 mM, *i.e.*, the concentration variation is *Δ* = 18 mM (Fig. 4C and gray solid line in Fig. 4E). In the experiment with competitor 2, this *Δ* had drastically decreased to just 3 mM when pseudo steady state was reached (red solid line in Fig. 4E). The concentration variation *Δ* was quantified by the theoretical kinetic model for increasing competitor 2 concentrations and tended to decrease (Fig. 4E and ESI Fig. 16†). In other words, co-phase separation protects the products from hydrolysis and buffers against fuel-driven oscillations. A reminiscent observation was recently reported in a population of Hela cells where phase separation was shown to buffer different expression levels.^55^

## Conclusions

In general, when metabolic reaction cycles compete for a common nutrient, both suffer because they need to share a common, scarce resource. In this work, we found a surprising behavior where competition can increase the success of one of the precursors, *i.e.*, it can survive longer or show reduced concentration oscillations in the presence of oscillatory fueling. The reason for this success relies on co-phase separation as a mechanism where droplets composed of both anhydrides create a protective environment. This behavior shows analogies to parasitic behavior in biology. The parasite competes with the host for resources and thereby decreases the lifetime of the host. Furthermore, the parasite exploits the protective environment of the host which increases its lifetime. This property could be crucial for the control of downstream chemical reactions of the competitors. Our results demonstrate that parasitic behavior can already emerge in a simple non-equilibrium system that can phase separate and is controlled by fuel-driven chemical reaction cycles. Our understanding of the underlying mechanism can be a step toward the design of more complex, synthetic life-like systems. In the future, we will explore how co-phase separation affects the selection of a large number of chemically active molecules.

## Materials and methods

### Materials

We purchased (*E*/*Z*)-2-buten-1-ylsuccinic anhydride (product) and (*E*/*Z*)-2-hexenyl-1-ylsuccinic anhydride (product of competitor 2) from TCI chemicals. Succinic acid (competitor 1), succinic anhydride (product of competitor 1), Nile Red, 1-ethyl-3-(3 dimethyl-aminopropyl)carbodiimide hydrochloride (EDC), trifluoroacetic acid (TFA) and 2-(*N*-morpholino)ethane sulfonic acid (MES buffer) were purchased from Sigma-Aldrich. All chemicals were used without any further purification unless otherwise indicated. High performance liquid chromatography (HPLC) grade acetonitrile (ACN) was purchased from VWR.

### Synthesis of the succinate precursors

(*E*/*Z*)-2-Buten-1-yl-succinic anhydride (product) and (*E*/*Z*)-2-hexenyl-1-ylsuccinic anhydride (product of competitor 2) were dispersed in 30 mL MQ water and stirred for 3 days. Subsequently, the reaction mixture was freeze-dried, and the corresponding succinates (precursor and competitor 2) were stored at −20 °C until further usage.

### HPLC

We used a ThermoFisher Dionex Ultimate 3000 analytical HPLC with a Hypersil Gold 250 × 4.6 mm C18 column (5 μm pore size) to monitor the concentration profiles of each reactant of the chemical reaction network. We prepared 1.0 mL samples according to the sample preparation protocol described above into a screw cap HPLC vial. Samples were injected directly from the HPLC vial without any further dilution. We injected 25 μL for the detection of the succinates and anhydrides and 1 μL for the detection of EDC. We used a UV/Vis detector at 220 nm for the detection. A linear gradient of MQ water : ACN with 0.1% TFA was used to separate the compounds. We used a linear gradient from 98 : 2 to 2 : 98 in 10 minutes followed by 2 minutes at 2 : 98 for the separation. The column was equilibrated for 2 minutes after each gradient. We performed calibration curves of the compounds in triplicates. Calibration values and retention times are given in ESI Tables S1 and S2.†

### Confocal fluorescence microscopy

We imaged the droplets using a Leica SP8 confocal microscope with a 63× oil immersion objective. Samples were prepared as described above but with 0.1 μM Nile Red added before EDC addition. We added 5 μL sample to a silicon grease reservoir on a PEG-coated glass slide covered with a 12 mm diameter coverslip. The samples were excited with a 543 nm laser and imaged at 580–700 nm.

### Droplet composition experiments

We prepared 5 mL samples as described above to guarantee a sufficient droplet phase volume after centrifugation. After the depletion of the fuel, the reaction mixture was centrifuged at 4 °C for 3 minutes (rpm = 5000). We used an Eppendorf pipette to take a 1 μL sample of the droplet pellet which we diluted in 200 μL ACN in a HPLC screw cap vial. We analyzed the ratio of product to product of competitor 2 with HPLC.

### Supernatant composition experiments

We prepared 1 mL samples as described above in 1.5 mL Eppendorf reaction vessels. After 16 minutes, the samples were centrifuged at 25 °C for 1 minute (rpm = 13 500). We directly analyzed the concentrations of the corresponding anhydrides in the supernatant of the sample with HPLC (see ESI Tables S4 and S5†).

### Calculation of respective concentrations inside the droplets

We calculated the corresponding anhydride concentrations inside the droplets using the relation *n*^I^ = *n*^total^ − *n*^II^ and their molecular volumes, respectively (see ESI Tables S3, S5–S7†). The superscripts I and II represent the droplet phase and aqueous bulk phase, respectively. The total volume of the droplets is represented as *V*^I^.

## Author contributions

J. B. and P. S. S. designed the experiments. P. S. S. and T. H. carried out the experiment. S. L., J. J. and C. A. W. worked out the theory. S. L. and J. J. performed the numerical studies and the fitting to the experimental data. P. S. S., S. L., J. B. and C. A. W. wrote the manuscript. All authors have given approval to the final version of the manuscript.

## Conflicts of interest

There are no conflicts to declare.

## Supplementary Material

SC-012-D1SC01106E-s001

## References

[cit1] Wang G., Liu S. (2020). ChemSystemsChem.

[cit2] Singh N., Formon G. J. M., De Piccoli S., Hermans T. M. (2020). Adv. Mater..

[cit3] Merindol R., Walther A. (2017). Chem. Soc. Rev..

[cit4] De S., Klajn R. (2018). Adv. Mater..

[cit5] Della Sala F., Neri S., Maiti S., Chen J. L., Prins L. J. (2017). Curr. Opin. Biotechnol..

[cit6] Kariyawasam L. S., Hossain M. M., Hartley C. S. (2020). Angew. Chem., Int. Ed..

[cit7] Ragazzon G., Prins L. J. (2018). Nat. Nanotechnol..

[cit8] Mishra A., Dhiman S., George S. J. (2021). Angew. Chem., Int. Ed..

[cit9] Rieß B., Wanzke C., Tena-Solsona M., Grötsch R. K., Maity C., Boekhoven J. (2018). Soft Matter.

[cit10] Tena-Solsona M., Rieß B., Grötsch R. K., Löhrer F. C., Wanzke C., Käsdorf B., Bausch A. R., Müller-Buschbaum P., Lieleg O., Boekhoven J. (2017). Nat. Commun..

[cit11] Kriebisch B. A. K., Jussupow A., Bergmann A. M., Kohler F., Dietz H., Kaila V. R. I., Boekhoven J. (2020). J. Am. Chem. Soc..

[cit12] Dai K., Fores J. R., Wanzke C., Winkeljann B., Bergmann A. M., Lieleg O., Boekhoven J. (2020). J. Am. Chem. Soc..

[cit13] Boekhoven J., Brizard A. M., Kowlgi K. N., Koper G. J., Eelkema R., van Esch J. H. (2010). Angew. Chem..

[cit14] Bal S., Das K., Ahmed S., Das D. (2019). Angew. Chem..

[cit15] Ogden W. A., Guan Z. (2020). ChemSystemsChem.

[cit16] Boekhoven J., Hendriksen W. E., Koper G. J. M., Eelkema R., van Esch J. H. (2015). Science.

[cit17] Jalani K., Das A. D., Sasmal R., Agasti S. S., George S. J. (2020). Nat. Commun..

[cit18] Dhiman S., Ghosh R., George S. J. (2020). ChemSystemsChem.

[cit19] Panzarasa G., Torzynski A. L., Sai T., Smith-Mannschott K., Dufresne E. R. (2020). Soft Matter.

[cit20] Tena-Solsona M., Wanzke C., Riess B., Bausch A. R., Boekhoven J. (2018). Nat. Commun..

[cit21] Tena-Solsona M., Janssen J., Wanzke C., Schnitter F., Park H., Rieß B., Gibbs J. M., Weber C. A., Boekhoven J. (2021). ChemSystemsChem.

[cit22] Donau C., Späth F., Sosson M., Kriebisch B. A. K., Schnitter F., Tena-Solsona M., Kang H.-S., Salibi E., Sattler M., Mutschler H., Boekhoven J. (2020). Nat. Commun..

[cit23] te Brinke E., Groen J., Herrmann A., Heus H. A., Rivas G., Spruijt E., Huck W. T. S. (2018). Nat. Nanotechnol..

[cit24] Wanzke C., Jussupow A., Kohler F., Dietz H., Kaila V. R. I., Boekhoven J. (2020). ChemSystemsChem.

[cit25] Maiti S., Fortunati I., Ferrante C., Scrimin P., Prins L. J. (2016). Nat. Chem..

[cit26] Solís Muñana P., Ragazzon G., Dupont J., Ren C. Z.-J., Prins L. J., Chen J. L.-Y. (2018). Angew. Chem., Int. Ed..

[cit27] Morrow S. M., Colomer I., Fletcher S. P. (2019). Nat. Commun..

[cit28] Grötsch R. K., Angı A., Mideksa Y. G., Wanzke C., Tena-Solsona M., Feige M. J., Rieger B., Boekhoven J. (2018). Angew. Chem., Int. Ed..

[cit29] Grötsch R. K., Wanzke C., Speckbacher M., Angı A., Rieger B., Boekhoven J. (2019). J. Am. Chem. Soc..

[cit30] Manna D., Udayabhaskararao T., Zhao H., Klajn R. (2015). Angew. Chem..

[cit31] van Ravensteijn B. G. P., Hendriksen W. E., Eelkema R., van Esch J. H., Kegel W. K. (2017). J. Am. Chem. Soc..

[cit32] Hossain M. M., Atkinson J. L., Hartley C. S. (2020). Angew. Chem., Int. Ed..

[cit33] Del Grosso E., Prins L. J., Ricci F. (2020). Angew. Chem., Int. Ed..

[cit34] Heinen L., Walther A. (2019). Sci. Adv..

[cit35] Zhang B., Jayalath I. M., Ke J., Sparks J. L., Hartley C. S., Konkolewicz D. (2019). Chem. Commun..

[cit36] Singh N., Lainer B., Formon G. J. M., De Piccoli S., Hermans T. M. (2020). J. Am. Chem. Soc..

[cit37] Heinen L., Heuser T., Steinschulte A., Walther A. (2017). Nano Lett..

[cit38] Zwicker D., Seyboldt R., Weber C. A., Hyman A. A., Jülicher F. (2017). Nat. Phys..

[cit39] Weber C. A., Zwicker D., Jülicher F., Lee C. F. (2019). Rep. Prog. Phys..

[cit40] Yang S., Schaeffer G., Mattia E., Markovitch O., Liu K., Hussain A. S., Ottelé J., Sood A., Otto S. (2021). Angew. Chem., Int. Ed..

[cit41] Post E. A., Fletcher S. P. (2020). Chem. Sci..

[cit42] Pross A., Khodorkovsky V. (2004). J. Phys. Org. Chem..

[cit43] Lotka A. J. (2002). J. Phys. Chem..

[cit44] Colomer I., Borissov A., Fletcher S. P. (2020). Nat. Commun..

[cit45] Altay M., Altay Y., Otto S. (2018). Angew. Chem., Int. Ed..

[cit46] Duim H., Otto S. (2017). Beilstein J. Org. Chem..

[cit47] Dadon Z., Wagner N., Alasibi S., Samiappan M., Mukherjee R., Ashkenasy G. (2015). Chem.–Eur. J..

[cit48] Altay Y., Altay M., Otto S. (2018). Chem.–Eur. J..

[cit49] Sadownik J. W., Mattia E., Nowak P., Otto S. (2016). Nat. Chem..

[cit50] Mattia E., Otto S. (2015). Nat. Nanotechnol..

[cit51] Post E. A. J., Fletcher S. P. (2020). Chem. Sci..

[cit52] Pascal R., Pross A. (2014). J. Syst. Chem..

[cit53] Wanzke C., Tena-Solsona M., Rieß B., Tebcharani L., Boekhoven J. (2020). Mater. Horiz..

[cit54] Krüger S., Weber C. A., Sommer J.-U., Jülicher F. (2018). New J. Phys..

[cit55] Klosin A., Oltsch F., Harmon T., Honigmann A., Jülicher F., Hyman A. A., Zechner C. (2020). Science.

