# Supplementary material for: Parasitic behavior in competing chemically fueled reaction cycles
Source: Chem Sci. 2021 Apr 28;12(21):7554–60. doi: 10.1039/d1sc01106e (PMC8171353; doi:10.1039/d1sc01106e)
Supplement: SC-012-D1SC01106E-s001 [file SC-012-D1SC01106E-s001.pdf]

Electronic Supplementary Material (ESI) for Chemical Science.
This journal is © The Royal Society of Chemistry 2021

Supporting Information

Parasitic Behavior in Competing Chemically Fueled Reaction Cycles

Patrick S. Schwarz,‡[1] Sudarshana Laha,‡[2,3] Jacqueline Janssen,[2,3] Tabea Huss,[1] Job Boekhoven,*[1,4] Christoph A. Weber*[2,3]

[1] Department of Chemistry, Technical University of Munich, Lichtenbergstrasse 4, 85748 Garching, Germany.

[2] Biological Physics, Max Planck Institute for the Physics of Complex Systems, Nöthnitzer Straße 38, 01187 Dresden, Germany.

[3] Center for Systems Biology Dresden, Pfotenhauerstrasse 108, 01307 Dresden, Germany.

[4] Institute for Advanced Study, Technical University of Munich, Lichtenbergstrasse 2a, 85748 Garching, Germany.

Supporting Information

In the Supporting Information, we provide Supporting Tables with the characterization of succinate derivatives and anhydrides and with the concentrations of the anhydrides in the aqueous and oil droplet phase, respectively which is used for the construction of phase diagram. We describe our kinetic model and compare the experimental data with the theory. We tabulate the reaction rate constants as obtained from fitting routines. We also calculate the droplet composition for different competitor 2 concentrations. Furthermore, we provide additional data for the co-phase separation mechanism, periodic fueling and buffer capacity.

1. Supporting Tables

Supporting Table S1: Characterization of succinate derivatives (xx = detection not possible).

Name	Structure	Mass calc. [g/mol]	Mass observed [g/mol]	Retention time [min]	Calibration value [mAU/mM]
**Competitor 1**	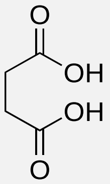	118.0 $C_4H_6O_4$	117.1 $[Mw-H]^-$	xx	xx
**Precursor**	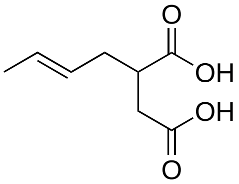	172.1 $C_8H_{12}O_4$	171.1 $[Mw-H]^-$	8.24	2.78
**Competitor 2**	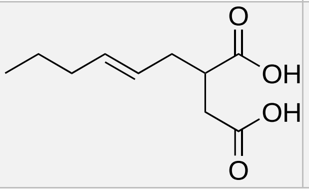	200.1 $C_{10}H_{16}O_4$	199.1 $[Mw-H]^-$	9.61	2.78

Supporting Table S2: Characterization of anhydrides.

Name	Structure	Mass calc. [g/mol]	Mass observed [g/mol]	Retention time [min]	Calibration value [mAU/mM]
**Product of competitor 1**	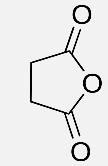	100.0 $C_4H_4O_3$	101.1 $[Mw+H]^+$	4.91	1.03
**Product**	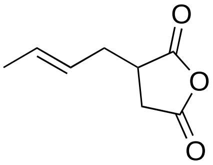	154.1 $C_8H_{10}O_3$	155.2 $[Mw+H]^+$	8.24	4.78
**Product of competitor 2**	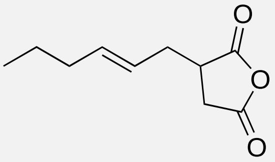	182.1 $C_{10}H_{14}O_3$	183.1 $[Mw+H]^+$	4.58	5.19

Supporting Table S3: Parameters required for ternary phase diagram construction.

Name	Density [g/cm^3]	Molar volume [cm^3/mol]	Molecular volume [cm^3]	Volume ratio with respect to water
**H_2O**	1.0	18.02	2.99E-23	1.0
**Product**	1.13	136.43	2.27E-22	7.57
**Product of Competitor 2**	1.07	170.30	2.83E-22	9.45

Supporting Table S4: Concentration outside of the droplet phase (solubilities) of the product for different initial competitor 2 concentrations.

Experiment	$c^{\mathrm{II,eq}}$(Product) [mmol/L]	Deviation [mmol/L]
**100 mM Precursor, 200 mM EDC**	27.80	0.15
**50 mM Precursor, 25 mM Competitor 2 100 mM EDC**	3.88	0.09
**50 mM Precursor, 50 mM Competitor 2 100 mM EDC**	1.91	0.25
**50 mM Precursor, 75 mM Competitor 2 100 mM EDC**	0.87	0.08
**50 mM Precursor, 100 mM Competitor 2 100 mM EDC**	0.61	0.05
**50 mM Precursor 100 mM Competitor 2**	0	0

Supporting Table S5: Concentration outside of the droplet phase (solubilities) of the product of competitor 2 for different initial competitor 2 concentrations.

Experiment	$c^{\mathrm{II,eq}}$(Product of Competitor 2) [mmol/L]	Deviation [mmol/L]
**100 mM Precursor, 200 mM EDC**	0	0
**50 mM Precursor, 25 mM Competitor 2 100 mM EDC**	1.97	0.07
**50 mM Precursor, 50 mM Competitor 2 100 mM EDC**	2.28	0.12
**50 mM Precursor, 75 mM Competitor 2 100 mM EDC**	1.94	0.10
**50 mM Precursor, 100 mM Competitor 2 100 mM EDC**	2.04	0.08
**50 mM Precursor 100 mM Competitor 2**	2.01	0.1

Supporting Table S6: Amount of substance and droplet volume for the product and product of competitor 2 for different initial competitor 2 concentrations.

Experiment	n^{I} (Product) [mmol]	n^{I} (Product of competitor 2) [mmol]	V^{I} [L]
**100 mM Precursor, 200 mM EDC**	5.85E-03	0	8.0E-07
**50 mM Precursor, 25 mM Competitor 2 100 mM EDC**	5.87E-03	1.30E-02	3.0E-06
**50 mM Precursor 50 mM Competitor 2 100 mM EDC**	4.81E-03	2.71E-02	5.3E-06
**50 mM Precursor, 75 mM Competitor 2 100 mM EDC**	3.98E-03	3.46E-02	6.4E-06
**50 mM Precursor, 100 mM Competitor 2 100 mM EDC**	3.20E-03	3.83E-02	7.0E-06
**50 mM Competitor 2 100mM EDC**	0	4.29E-02	7.3E-06

Supporting Table S7: Concentrations inside the droplets for the product and product of competitor 2 for different initial competitor 2 concentrations.

Experiment	$c^{\mathrm{I,eq}}$ (Product) [mmol/L]	$c^{\mathrm{I,eq}}$ (Product of competitor 2) [mmol/L]
**100 mM Precursor, 200 mM EDC**	7.32E+03	0
**50 mM Precursor, 25 mM Competitor 2 100 mM EDC**	1.95E+03	4.30E+03
**50 mM Precursor 50 mM Competitor 2 100 mM EDC**	8.37E+02	5.19E+03
**50 mM Precursor, 75 mM Competitor 2 100 mM EDC**	6.64E+02	5.33E+03
**50 mM Precursor, 100 mM Competitor 2 100 mM EDC**	4.66E+02	5.49E+03
**50 mM Competitor 2 100mM EDC**	0	5.87E+03

2. Theoretical kinetic model for co-phase separation and chemical reactions competing for fuel

Fuel driven chemical reactions

Our system is composed of four different types of molecules: fuel, succinate derivatives, intermediate and anhydride molecules. The fuel molecule EDC is abbreviated by the letter F. We consider three types of succinate derivatives which we refer to as precursor (A_P), competitor 1 (A_{C_1}) and competitor 2 (A_{C_2}), respectively, and abbreviate each derivative as A_i with $i = P, C_1, C_2$. The succinate derivatives used experimentally are (E/Z)-2-buten-1-ylsuccinic acid (precursor), succinic acid (competitor 1) and (E/Z)-2-hexen-1-ylsuccinic acid (competitor 2). The intermediate molecule, AF_i, is composed of fuel and the respective succinate derivative. There are three corresponding anhydrides, abbreviated as B_i, and referred to as product, product of competitor 1 or product of competitor 2. The anhydrides used experimentally are (E/Z)-2-buten-1-ylsuccinic anhydride (product), succinic anhydride (product of competitor 1) and (E/Z)-2-hexen-1-ylsuccinic anhydride (product of competitor 2). In our systems, the solvent is water (w).

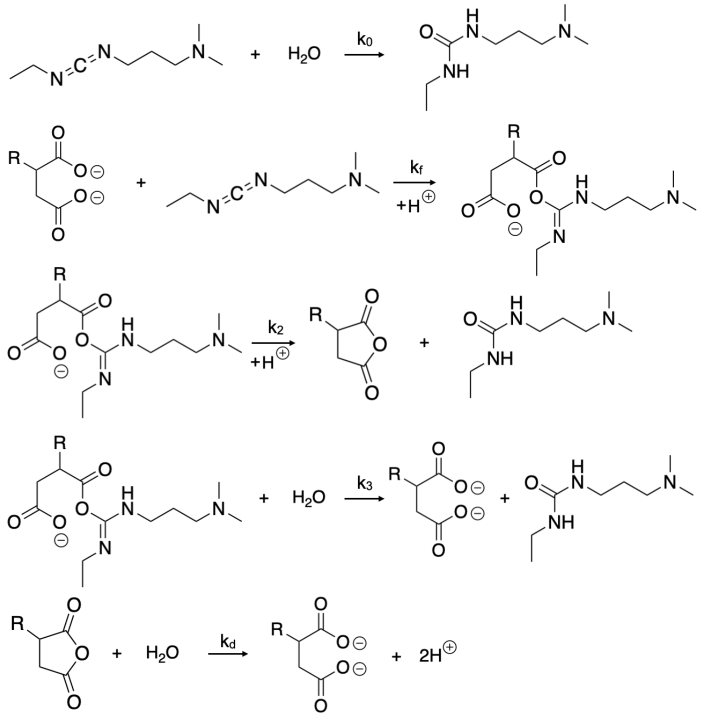

Figure 1: Chemical reactions considered in the kinetic model. Reaction (0) corresponds to the direct hydrolysis of EDC to waste, EDU. **Reaction (1)** shows the activation reaction of the succinate derivative with EDC to form the intermediate molecule. **Reaction (2)** is the intramolecular anhydride formation reaction. **Reaction (3)** depicts the direct hydrolysis of the intermediate molecule. **Reaction (4)** shows the hydrolysis of the anhydride to the initial succinate derivative.

The fuel F can undergo two chemical reactions: F can get slowly hydrolysed to waste W with a rate constant k_0 (reaction 0, Eq. (1a)), or, the fuel F drives the transition from the succinate derivative A_i to the intermediate AF_i with a chemical flux that is proportional to the fuel concentration c_F with a rate constant $k_{f,i}$ (reaction 1, second order chemical reaction). This intermediate molecule, AF_i can spontaneously hydrolyse back to A_i with a rate constant $k_{3,i}$, or irreversibly turn over to the anhydride B_i (reactions 1-3, Eq. (1b)). The turn-over to the anhydride (reaction 2) occurs spontaneously as an intramolecular reaction with a rate constant $k_{2,i}$ (first-order reaction). In aqueous media, this anhydride hydrolyses and thereby turns over to the initial succinate derivative (Eq. (1c)), which we shortly refer to as deactivation step. Considering a constant pH and approximately dilute conditions relative to water, deactivation follows a first order chemical reaction with a deactivation rate constant $k_{d,i}$. All reactions (0)-(4) as shown in Fig.1, can be summarised by the following reaction schemes:

$$\text{Reaction 0:} \quad F \xrightarrow{k_0} W \,, \tag{1a}$$

$$\text{Reactions 1-3:} \quad A_i \underset{k_{3,i}}{\overset{k_{f,i}\, c_F}{\rightleftharpoons}} AF_i \xrightarrow{k_{2,i}} B_i \,, \tag{1b}$$

$$\text{Reaction 4:} \quad B_i \xrightarrow{k_{d,i}} A_i \,. \tag{1c}$$

Co-phase separation of anhydride molecules

At concentrations larger than their respective solubilities (Supporting Table S4, S5), each anhydride B_i (product and product of competitor 2) can phase separate from the water-rich solvent w and form oil-like droplets that are rich in anhydride. Since fuel F, the succinate derivatives A_i (precursor and competitor 2) and intermediate molecules AF_i, are well soluble in water, the droplets dominantly contain the rather hydrophobic anhydrides B_i with concentrations inside compared to the outside similar to oil drops in water. Here, we study the interplay of the product (P) and the product of competitor 2 (C_2), which co-phase separate. To estimate the co-phase separation properties of these two anhydrides with respect to the solvent, we consider the limit of fuel excess. In this case, the system is mostly composed of the two anhydrides, solvent and fuel. Due to the hydrophilic property of the fuel, we neglect its effects on phase separation. Thus, we can determine a ternary phase diagram for the remaining three molecules (product and product of competitor 2, and solvent w). As a model for this phase diagram, we consider a ternary, incompressible Flory-Huggins free energy density,

$$f = \frac{k_{\mathrm{B}}T}{\nu_w} \left(\sum_i \frac{\phi_i}{r_i} \ln \phi_i + \sum_{i,j:i\neq j} \frac{\chi_{ij}}{2}\, \phi_i \phi_j \right) \,, \tag{2}$$

where the summation index $i, j = P, C_2, w$, runs over all three types of molecules, i.e., product, product of competitor 2 and water as the solvent, respectively. Moreover, $r_i = \nu_i/\nu_w$, where ν_i is the molecular volume of component i and ν_w is the molecular volume of water. Incompressibility implies that all molecular volumes are constant and that the volume fractions ϕ_i obey, $1 - \phi_w = \phi_P + \phi_{C_2}$. These volume fractions are related to concentrations, $c_i = \phi_i/\nu_i$. Both hetero- and homotypic interactions between molecules i and j are accounted for by the interaction parameter χ_{ij}. Our

model for the phase diagram has 5 parameters: the molecular volumes of the two anhydrides, ν_P and ν_{C_2}, the interaction parameters between each anhydride and solvent, $\chi_{P,w}$ and $\chi_{C_2,w}$, and the effective interaction parameter between the two anhydrides, χ_{P,C_2}. The ternary phase diagram is obtained from the free energy density (Eq. (2)) by a Maxwell construction, where the volume fractions in droplet phase (I), $\phi_P^{\mathrm{I,eq}}$ and $\phi_{C_2}^{\mathrm{I,eq}}$, coexist with the volume fractions in aqueous phase (II), $\phi_P^{\mathrm{II,eq}}$ and $\phi_{C_2}^{\mathrm{II,eq}}$. The volume fractions fulfil the equilibrium conditions for phase coexistence:

$$\bar{\mu}_P(\phi_P^{\mathrm{I,eq}}, \phi_{C_2}^{\mathrm{I,eq}}) = \bar{\mu}_P(\phi_P^{\mathrm{II,eq}}, \phi_{C_2}^{\mathrm{II,eq}}) \, , \tag{3a}$$

$$\bar{\mu}_{C_2}(\phi_P^{\mathrm{I,eq}}, \phi_{C_2}^{\mathrm{I,eq}}) = \bar{\mu}_{C_2}(\phi_P^{\mathrm{II,eq}}, \phi_{C_2}^{\mathrm{II,eq}}) \, , \tag{3b}$$

$$\Pi(\phi_P^{\mathrm{I,eq}}, \phi_{C_2}^{\mathrm{I,eq}}) = \Pi(\phi_P^{\mathrm{II,eq}}, \phi_{C_2}^{\mathrm{II,eq}}) \, , \tag{3c}$$

where the exchange chemical potentials $\bar{\mu}_i = \nu_i \partial f / \partial \phi_i$ and the osmotic pressure $\Pi = -f + \sum_i \mu_i \phi_i / \nu_i$ can be calculated from the free energy density (Eq. (2)), and $i = P, C_2$. In the Maxwell construction for a ternary, incompressible mixture, we have two more conditions, namely the conservation laws for the total volume fraction for each anhydride, $\bar{\phi}_i = \left(V^{\mathrm{I}} \phi_i^{\mathrm{I,eq}} + (V - V^{\mathrm{I}}) \phi_i^{\mathrm{II,eq}} \right) / V$. The equilibrium volume fractions in both phases for each anhydride ($\phi_i^{\mathrm{I,eq}}$, $\phi_i^{\mathrm{II,eq}}$), together with the droplet volume V^{I}, give us five unknowns and we have five equations determining these unknowns.

Obtaining interaction parameters from the experimental phase diagram

Using the observed molar masses (Supporting Table S1), we find for the product (P) and the product of competitor 2 (C_2), $m_P/m_w = 8.56$ and $m_{C_2}/m_w = 10.11$, respectively. To fit the experimental phase diagram, we used the molecular masses relative to water as initial guesses for the fractions of the molecular volumes, i.e., $r_i = \nu_i/\nu_w \simeq m_i/m_w$, where m_i denotes the molecular mass of molecule i. Given the experimental equilibrium concentrations of the anhydride molecules i ($\phi_i^{\mathrm{I,eq}}$, $\phi_i^{\mathrm{II,eq}}$), in their respective binary system, i.e., product (P) with solvent (w) and product of competitor 2 (C_2) with solvent (w), we can solve Eq. (3) for $\chi_{P,w}, \nu_P, \chi_{C_2,w}$ and ν_{C_2}. In the main text $\phi^{\mathrm{II,eq}}$ is referred to as c_{out}.

Thus, for the fraction of molecular volumes $r_i = \nu_i/\nu_w$, we obtain $r_P = 6.44$ and $r_{C_2} = 8.35$ which are in good agreement with the mass fraction (see previous paragraph). The obtained interaction parameters in the respective binary system are $\chi_{P,w} = 1.63$ and $\chi_{C_2,w} = 1.76$ (in units of $k_{\mathrm{B}}T$). Keeping these interaction parameters and the molecular volume fractions fixed, we have only one undetermined parameter left, namely χ_{P,C_2}. This parameter is obtained by finding the best agreement with the binodal lines and tie line slopes. Very good agreement is obtained for the value $\chi_{P,C_2} = 0$, see Fig.2. A zero χ_{P,C_2} parameter is consistent with the homotypic interactions and heterotypic interactions between the two anhydrides being approximately of the same magnitude; a scenario that is reasonable due to similarity of the molecular structures of the two anhydrides.

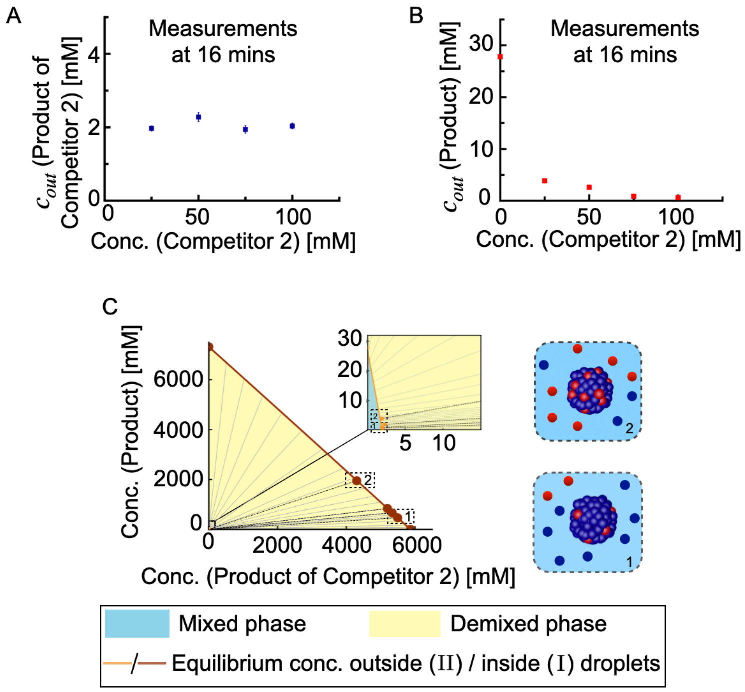

Figure 2: Ternary phase diagram. A) The concentration of the product of competitor 2 in the aqueous phase as function of competitor 2 concentration. The value remains nearly constant around 2 mM. B) The concentration of the product in the aqueous phase as function of competitor 2 concentration. In the absence of competitor 2, the value is 27.8 mM (solubility) and it decreases with increasing competitor 2 concentration. The measurements are performed at 16 mins into the reaction cycle. C) Linear representation of the ternary phase diagram, highlighting that it spans over 3 orders of magnitude in concentration values. The circles denote the experimental data points and the solid lines represent theoretically determined binodal lines and tie lines obtained from solving Eq. (3). The dashed black lines are the experimental tie lines. In the inset, we show the equilibrium concentrations in the aqueous phase (II). The compositions of the aqueous phase and oil droplet phase differ depending on which tie line the total anhydride concentrations lie.

Kinetic model for phase separation of products and fuel-driven chemical reactions

To describe the kinetics of all the reacting molecules in the system, we introduce the concentrations of the two anhydrides (product P and product of competitor 2 C_2) inside the droplet phase (I) as $c^{\mathrm{I}}_{B_i}$, where $i = P$, C_2 and the concentrations of all other molecules outside the droplet as c_j, where $j = F, A_i, AF_i, B_i$ denotes fuel, succinate derivates (precursor and competitor 2), intermediate molecules, and anhydrides (product and product of competitor 2), respectively. Within our model, these components can undergo chemical reactions as described in Eq. (1), and the anhydrides (product and product of competitor 2) can phase-separate. Supported by the experimental observation that droplets form very quickly on the experimentally relevant time scales of minutes, we propose a simplified model for the total concentrations within the demixed region of the phase diagram. This model relies on fast phase separation and partitioning kinetics of the anhydrides

relative to their chemical reactions. Specifically, this model is valid if the inter-droplet distances do not exceed the reaction-diffusion length scale $\sqrt{D_i/k_{d,i}}$ for both anhydrides. Using experimental values, this reaction-diffusion length scale is in the order of a few hundreds μm, while inter-droplet distances are about a few tenths of μm, supporting the validity of this approximation. Thus, the reaction-partitioning equations for the kinetics of all molecules outside the droplets (II) and the kinetics of the total anhydride concentrations read:

$$\partial_t c_F = - \sum_{i=P,C_2} k_{f,i}\, c_F\, c_{A_i} - k_0\, c_F \, , \tag{4a}$$

$$\partial_t c_{A_i} = -k_{f,i}\, c_F\, c_{A_i} + k_{3,i}\, c_{AF_i} + k_{d,i}\, c_{B_i} \, , \tag{4b}$$

$$\partial_t c_{AF_i} = k_{f,i}\, c_F\, c_{A_i} - (k_{3,i} + k_{2,i})\, c_{AF_i} \, , \tag{4c}$$

$$\partial_t c_{B_i} = k_{2,i}\, c_{AF_i} - k_{d,i}\, c_{B_i} \, , \tag{4d}$$

$$\partial_t \bar{c}_{B_i} = (1 - V^{\mathrm{I}}/V)\, (k_{2,i}\, c_{AF_i} - k_{d,i}\, c_{B_i}) \, . \tag{4e}$$

Under the valid assumption of fast partitioning kinetics compared to the slow chemical reactions outside the droplets, the total concentrations of the two anhydrides, $\bar{c}_{B_i}(t)$ determine their equilibrium concentrations (via Maxwell construction using Eq. (2) in Eqs. (3)) and the volume of the dense phase at each time point t:

$$\text{Maxwell construction:} \quad \bar{c}_{B_i}(t) \to c_{B_i}^{\mathrm{I,eq}}(t),\, c_{B_i}^{\mathrm{II,eq}}(t) \, , \tag{4f}$$

$$\text{Volume dense phase:} \quad V^{\mathrm{I}}(t) = V \frac{\bar{c}_{B_i}(t) - c_{B_i}^{\mathrm{II,eq}}(t)}{c_{B_i}^{\mathrm{I,eq}}(t) - c_{B_i}^{\mathrm{II,eq}}(t)} \, , \tag{4g}$$

for $i = P$, C_2 and V is the total volume of the system which is a constant ($V =$ 1.05 mL).

In summary, in our model which considers the limit of fast phase separation compared to chemical reactions, the chemical reactions affect the total concentrations of both anhydrides, leading to instantaneous changes of their respective equilibrium concentrations. These equilibrium concentrations varying with time modify the concentration levels in both phases (I and II) via partitioning and thereby in turn affect the chemical reactions. Due to this feedback between chemical reactions and phase separation, the total concentrations move on a specific path in the two dimensional phase diagram which is unique to the initial succinate derivative and fuel concentrations (see Fig. 3E in main text). The total concentrations of the anhydrides, i.e., $\bar{c}_{B_i}(t)$, can also lie outside the demixed region during the reaction cycle, implying that phase separation is absent and the system is homogeneous (mixed phase). In that case, there is no partitioning and $V^{\mathrm{I}} = 0$, such that only the kinetic equations, i.e., Eqs. (4a) (4b) (4c), and Eq. (4e), are to be solved.

When we study the competition system between product and product of competitor 1, we also apply the aforementioned approach to the case where phase separation of anhydrides is absent and the system is always homogeneous.

3. Obtaining reaction rate constants for the kinetic model

We study three succinate derivatives, which we label as P, C_1 and C_2 and their corresponding anhydrides. We fit the experimental measurements with the kinetic traces of each of the reaction

cycles to determine the rate constants (Figs.3, 4 and 5).

Systems with one succinate derivative

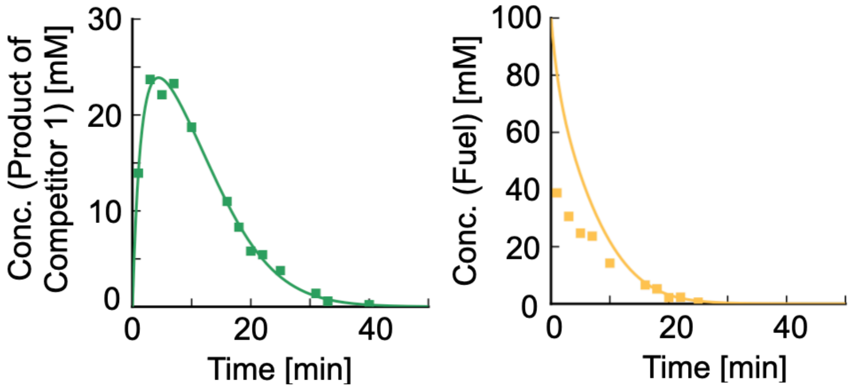

Figure 3: First order deactivation and short lifetime of product of competitor 1. 50 mM competitor 1 fuelled with 100 mM EDC. The two curves, corresponding to the time trace of the product of competitor 1 and fuel concentration respectively, are globally fitted to obtain the reaction rate constants. The concentration profile of product of competitor 1 shows an exponential decay as it is not able to phase separate due to its high solubility of roughly 3000 mM. Markers represent HPLC data; solid lines represent data calculated using the theoretical kinetic model.

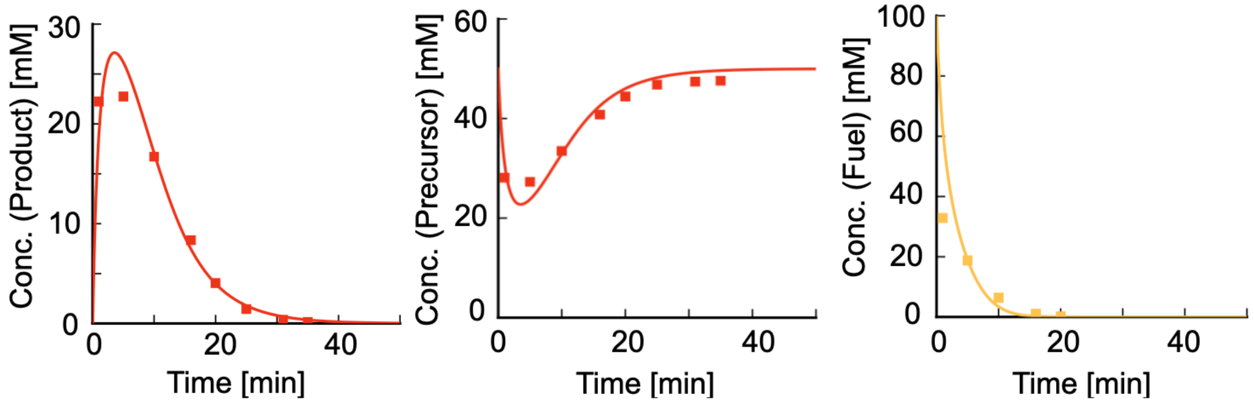

Figure 4: First order deactivation and short lifetime of product. 50 mM precursor fuelled with 100 mM EDC. The three curves, corresponding to the time trace of the product, precursor and fuel concentration respectively, are globally fitted to obtain the reaction rate constants. The product concentration profile shows an exponential decay as it is not able to phase separate due to its high solubility of roughly 27 mM. Markers represent HPLC data; solid lines represent data calculated using the theoretical kinetic model.

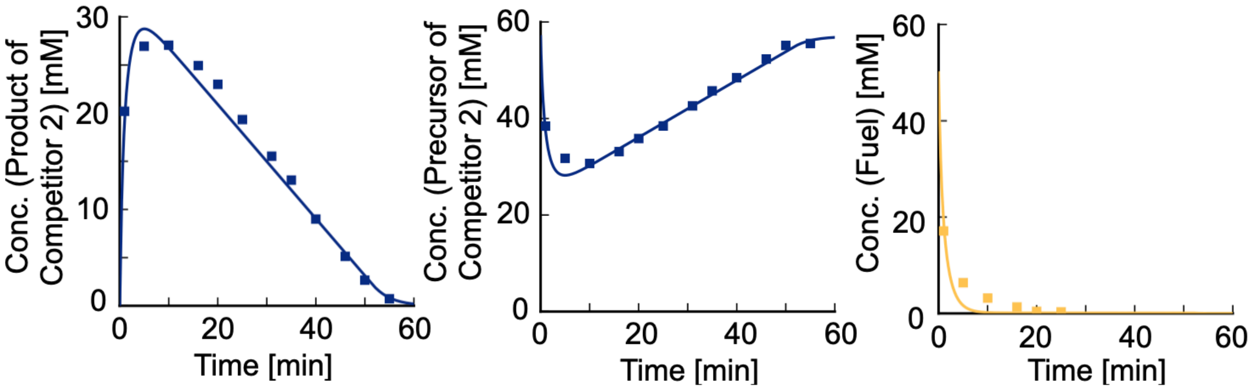

Figure 5: Zeroth order deactivation and long lifetime of product of competitor 2. 50 mM competitor 2 fuelled with 50 mM EDC. The three curves, corresponding to the time trace of the product of competitor 2, competitor 2 and fuel concentration respectively, are globally fitted to obtain the reaction rate constants. The concentration profile of product of competitor 2 shows a linear decay as it is able to phase separate due to its low solubility of roughly 2 mM. Markers represent HPLC data; solid lines represent data calculated using the theoretical kinetic model. The concentration of competitor 2 had to be re-adjusted to 57 mM in the theoretical kinetic model for the fitting procedure due to inaccuracies in the stock solution.

System with two competing succinate derivatives

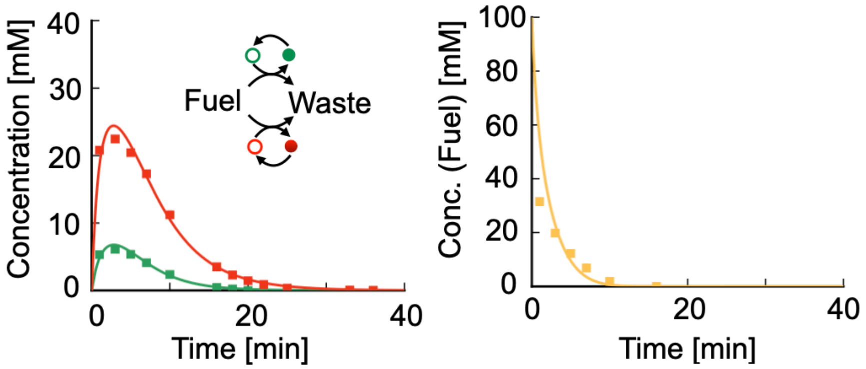

Figure 6: First order deactivation for the product and product of competitor 1. 50 mM precursor and 50 mM competitor 1 fuelled with 100 mM EDC. The three curves, corresponding to the time trace of two anhydrides and fuel concentration respectively, are globally fitted to obtain the reaction rate constants. The concentration profiles of product and product of competitor 1 both show exponential decay as neither is able to phase separate, and competition for fuel results in reduced yields for both anhydrides. Markers represent HPLC data; solid lines represent data calculated using the theoretical kinetic model.

Having obtained the reaction rate constants from the above fits in Fig. 6, we proceed to see how the traces change with changing competitor 1 (C_1) concentration and using fixed precursor (P) and EDC concentrations (Fig.7).

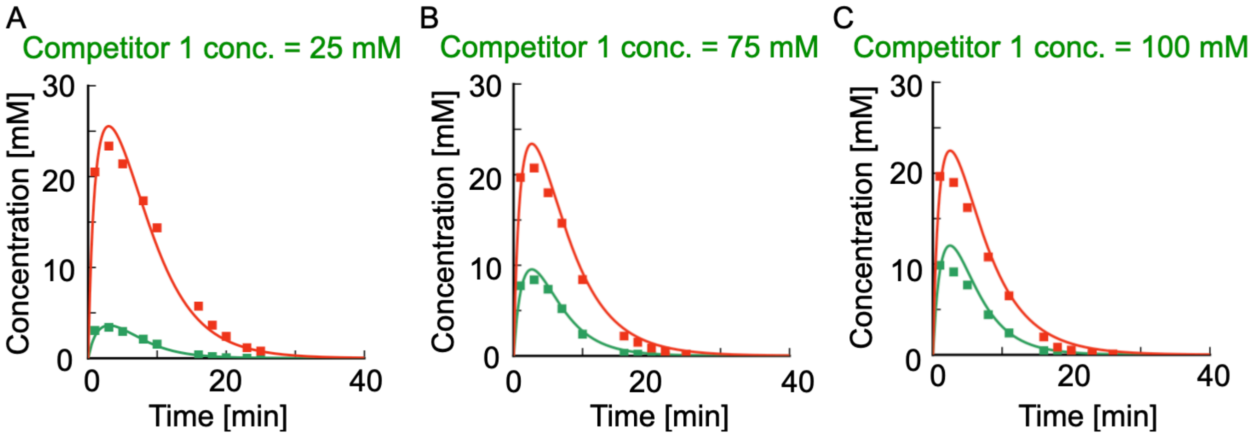

Figure 7: Competitor 1 reduces the yield and lifetime of the product. A) 25 mM C_1 , B) 75 mM C_1 , C) 100 mM C_1 , and 50 mM P fuelled with 100 mM EDC. Increasing the competitor 1 concentration reduces the maximum yield of the product and also its lifetime. Markers represent HPLC data; solid lines represent data calculated using the theoretical kinetic model.

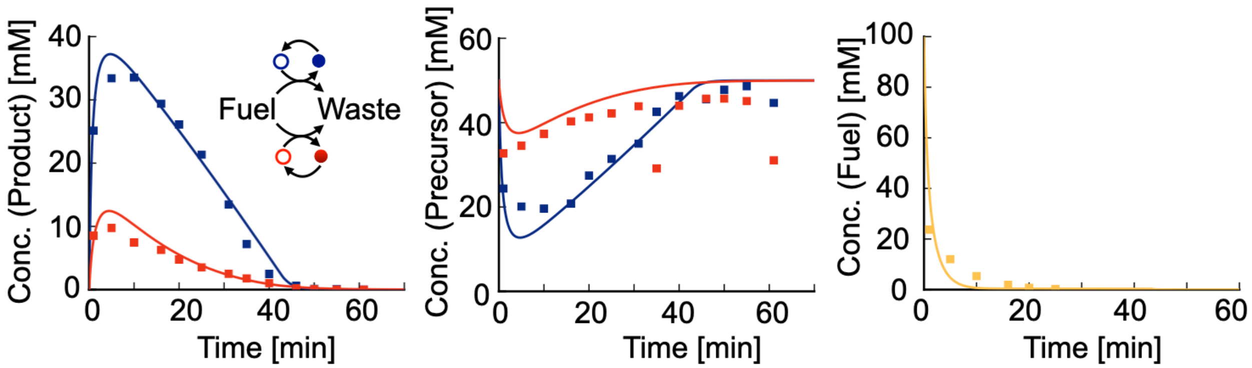

Figure 8: Non-linear and linear deactivation for the product and product of competitor 2 respectively. 50 mM precursor (P) and 50 mM competitor 2 (C_2) fuelled with 100 mM EDC. We globally fit five curves corresponding to the time trace of two anhydrides, two succinate derivatives and fuel concentration, respectively, to obtain the reaction rate constants. The two outlier points in the precursor concentration trace are omitted however. The linear decay of product of competitor 2 shows that it phase separates and the non-linear decay of the product implies that it partitions in the droplets, which we refer to as co-phase separation. Markers represent HPLC data; solid lines represent data calculated using the theoretical kinetic model.

Having obtained the reaction rate constants from the above fits in Fig. 8, we proceed to see how the traces change with changing competitor 2 (C_2) concentration and using fixed precursor (P) and EDC concentrations (Fig.9).

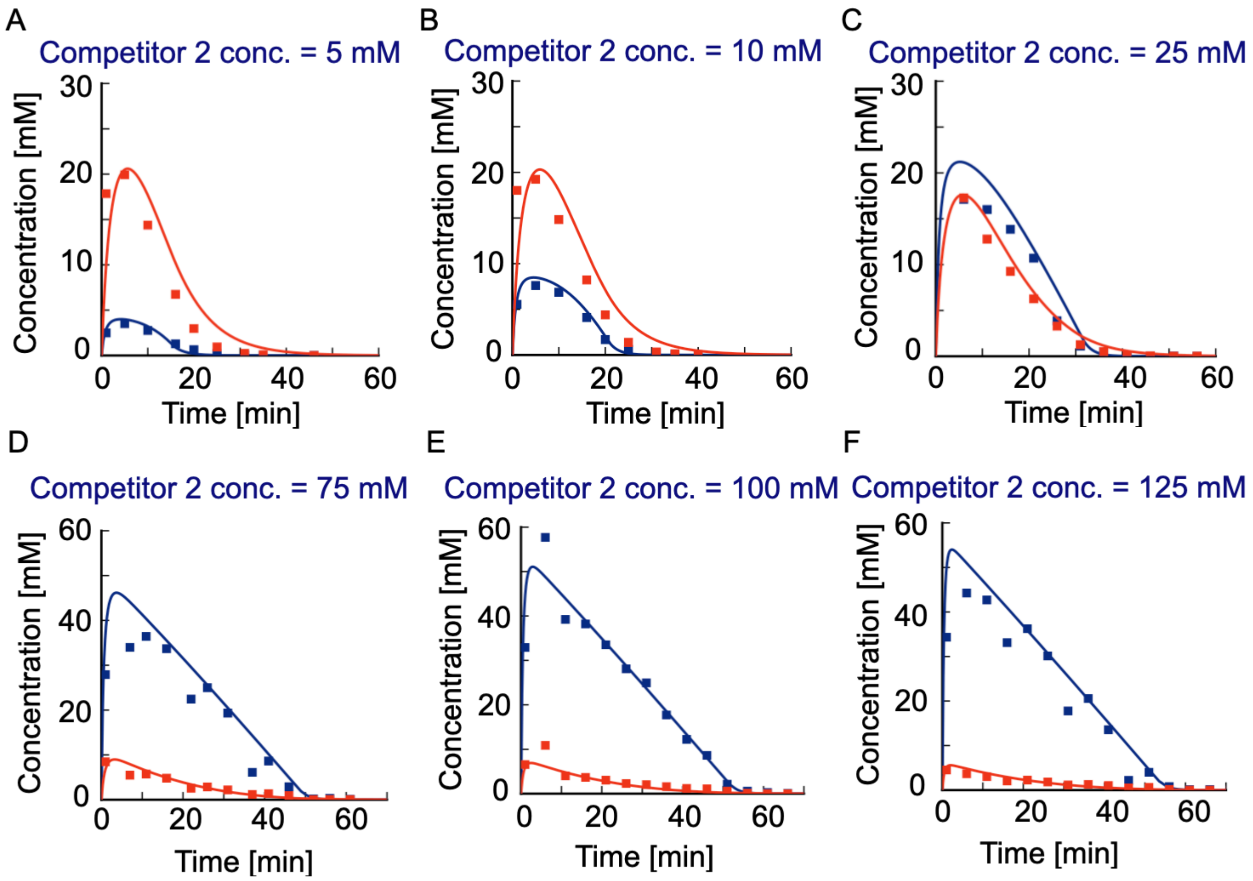

Figure 9: Competitor 2 reduces the yield but increases lifetime of the product. A) 5 mM C_2 , B) 10 mM C_2 , C) 25 mM C_2 , D) 75 mM C_2 , E) 100 mM C_2 , F) 125 mM C_2 and 50 mM P fuelled with 100 mM EDC. Increasing competitor 2 concentration reduces the maximum yield of the product, but prolongs its lifetime, allowing us to label competitor 2 as ***host*** and precursor as ***parasite***. Markers represent HPLC data; solid lines represent data calculated using the theoretical kinetic model.

Summary of reaction rate constants

Name	Condition	k_0 [s^{-1}]	k_f [$M^{-1}*s^{-1}$]	k_2 [s^{-1}]	k_3 [s^{-1}]	k_d [s^{-1}]
**Competitor 1**	**Single**	1.35e-5	0.085	0.52	0.11	3.7e-3
	**w/ Precursor**		0.037	0.58	0.23	4.2e-3
**Precursor**	**Single**	1.35e-5	0.20	0.63	0.52	2.7e-3
	**w/ Competitor 1**		0.21	0.37	0.28	3.2e-3
	**w/ Competitor 2**		0.10	0.30	0.32	1.0 - 2.3e-3
**Competitor 2**	**Single**	1.35e-5	0.35	0.50	0.26	4.9 - 6.8e-3
	**w/ Precursor**		0.35	0.50	0.26	8.8e-3

Figure 10: Reaction rate constants. Readout for the chemical reaction rate constants used in the kinetic model as obtained from the global fits.

We summarise the reaction rate constants obtained from fits as mentioned above (Figs.3, 4, 5, 6 and 8). We observed that in general there are only little differences in the deactivation rate constants of the single precursor system and to the system where two components compete about fuel. However, a noticeable effect is the reduction in the activation pathway rate constant, primarily k_f of competitor 1 (C_1) in the competition system with precursor (P) and that of the precursor (P) in the competition system with competitor 2 (C_2). For both single and competition studies we keep the solubilities of the product and product of competitor 2 unchanged to values, 2.01 mM and 27.8 mM, respectively. We thus assumed that competition for the fuel affects the availability of fuel for specific succinate derivatives. For periodic fueling studies, the lower value k_d was used for the product. For the Fig. 5 we use the lower value of k_d and for Fig.1F in the main text, we use the higher value of k_d for product of competitor 2 as it was obtained from fitting routine.

4. Lifetime and half lifetime measurements for the product

The lifetime of the anhydrides is defined phenomenologically by threshold concentrations. The lifetime corresponds to the time period when the anhydride concentration is above this threshold. A threshold concentration can lose robustness against experimental measurements if it lies in the tailing regime of an exponential decay. It also becomes an injudicious choice if set to a high concentration value which is unachieved during the course of the experiment. Therefore, we choose threshold concentration values as described below such that the aforementioned difficulties are circumvented.

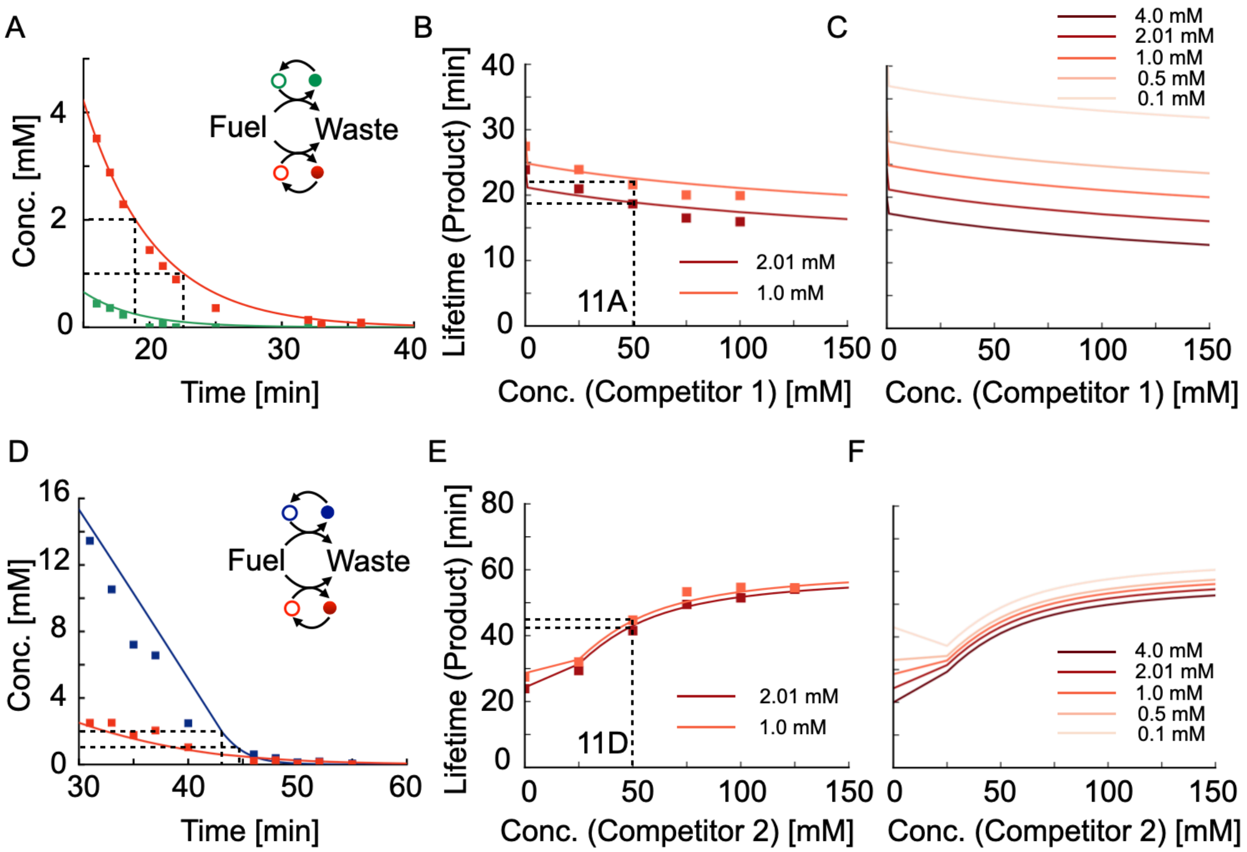

Figure 11: Co-phase separation allows longer survival of the product due to protection inside droplets from hydrolysis. A) The anhydride concentration profiles in the low concentration regime when 50 mM precursor and 50 mM competitor 1 compete for 100 mM fuel. The black dashed lines denote how the lifetimes are measured corresponding to threshold conc. values 2.01 mM and 1.0 mM. B) The lifetime of product measured for threshold concentrations of 2.01 mM and 1.0 mM. C) Trend of lifetime is robust against different threshold choices (4 mM, 2.01 mM, 1 mM, 0.5 mM and 0.1 mM). The lifetime of product decreases with increasing competitor 1 concentration. D) The anhydride concentration profiles in the low concentration regime when 50 mM precursor and 50 mM competitor 2 compete for 100 mM fuel. The black dashed lines denote how the lifetimes are measured corresponding to threshold conc. values 2.01 mM and 1.0 mM. E) The lifetime of product measured for threshold concentrations of 2.01 mM and 1.0 mM. F) Trend of lifetime is robust against different threshold choices (4 mM, 2.01 mM, 1 mM, 0.5 mM and 0.1 mM). The lifetime of product increases with increasing competitor 2 concentration. Markers represent lifetimes calculated using HPLC data; solid lines represent data calculated using the theoretical kinetic model.

We have performed two sets of competition experiments where precursor competes with competitor 1 and competitor 2, respectively. The important difference between the two competitors is that competitor 2 has a significantly lower solubility of about 2 mM compared to competitor 1 which has solubility of roughly 3000 mM, and therefore the former is capable of phase separation in the

working concentration ranges. The lifetime measurements, corresponding to Fig. 3B in the main text, are done by tracking a threshold concentration of 2.01 mM for the product. Similarly, corresponding to Fig. 3E in the main text, the lifetime measurement of the product is done by tracking the threshold concentration 2.01 mM for the product of competitor 2, as it highlights the lifetime of the droplets. We believe that the lifetimes of both the host (product of competitor 2) and parasite (product) are the same in this case. However this form of measurement is undefined for small concentration values of competitor 2 since droplets are short-lived and the product survives after droplet dissolution. In these concentration ranges we track the threshold concentration for the product instead of the product of competitor 2. We therefore choose to measure the half lifetime of the product which has a robust definition in either competition system.

The half lifetime is measured as the time difference between the time points of the maximum value and the half maximum value of the product concentration, respectively. In the experimental data, we locate the maximum value of the product concentration, calculate its halved value and determine the time point by linearly interpolating between the immediate high and low value around the half maximum. Refer to Fig. 12 for visualisation.

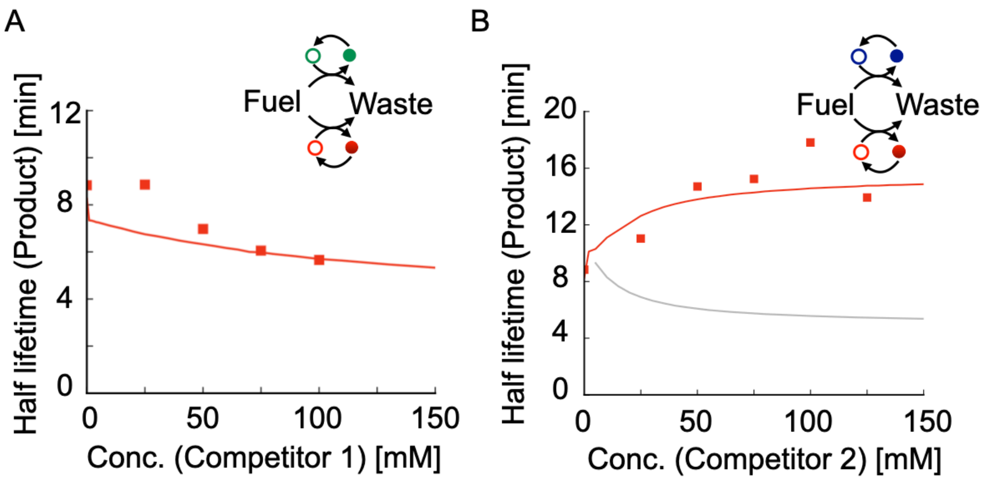

Figure 12: Half lifetime of product as a measure of survival in presence of competition. A) Half lifetime of product as a function of competitor 1 concentration. The half lifetime reduces due to competition for fuel in the system. B) Half lifetime of product as a function of competitor 2 concentration. The half lifetime increases due to protection in droplets from hydrolysis, but approaches saturation owing to a fixed fuel concentration of 100mM. The gray line denotes that in absence of co-phase separation in the competition system with competitor 2, the half lifetime would decrease solely due to competition for the fuel. Markers represent half lifetime calculated using HPLC data; solid lines represent data calculated using the theoretical kinetic model.

5. Composition of droplets

We quantified the percentage of the droplet material composed of the product for three different conditions with increasing competitor 2 concentration.

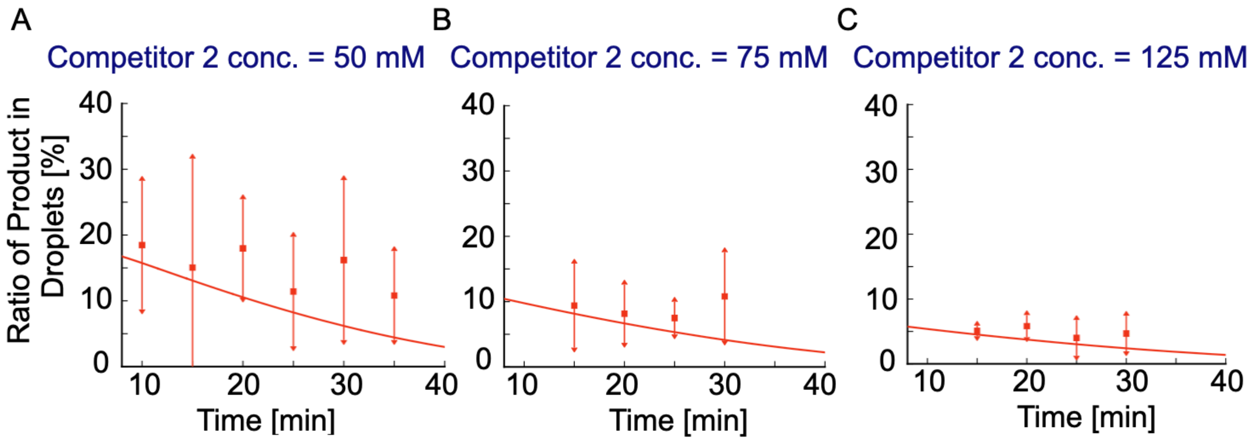

Figure 13: Reduction in composition of the product (parasite) in the droplets with increasing product of competitor 2 (host) concentration. A) 50 mM, B) 75 mM, C) 125 mM, respectively. Increasing concentration of the host reduces the maximum concentration of the parasite in the droplets. Also the maximum value of product monotonically decreases with time as the total volume of droplets keeps decreasing. Markers represent ratio calculated using HPLC data; solid lines represent data calculated using the theoretical kinetic model.

6. Mechanism of Co-phase separation

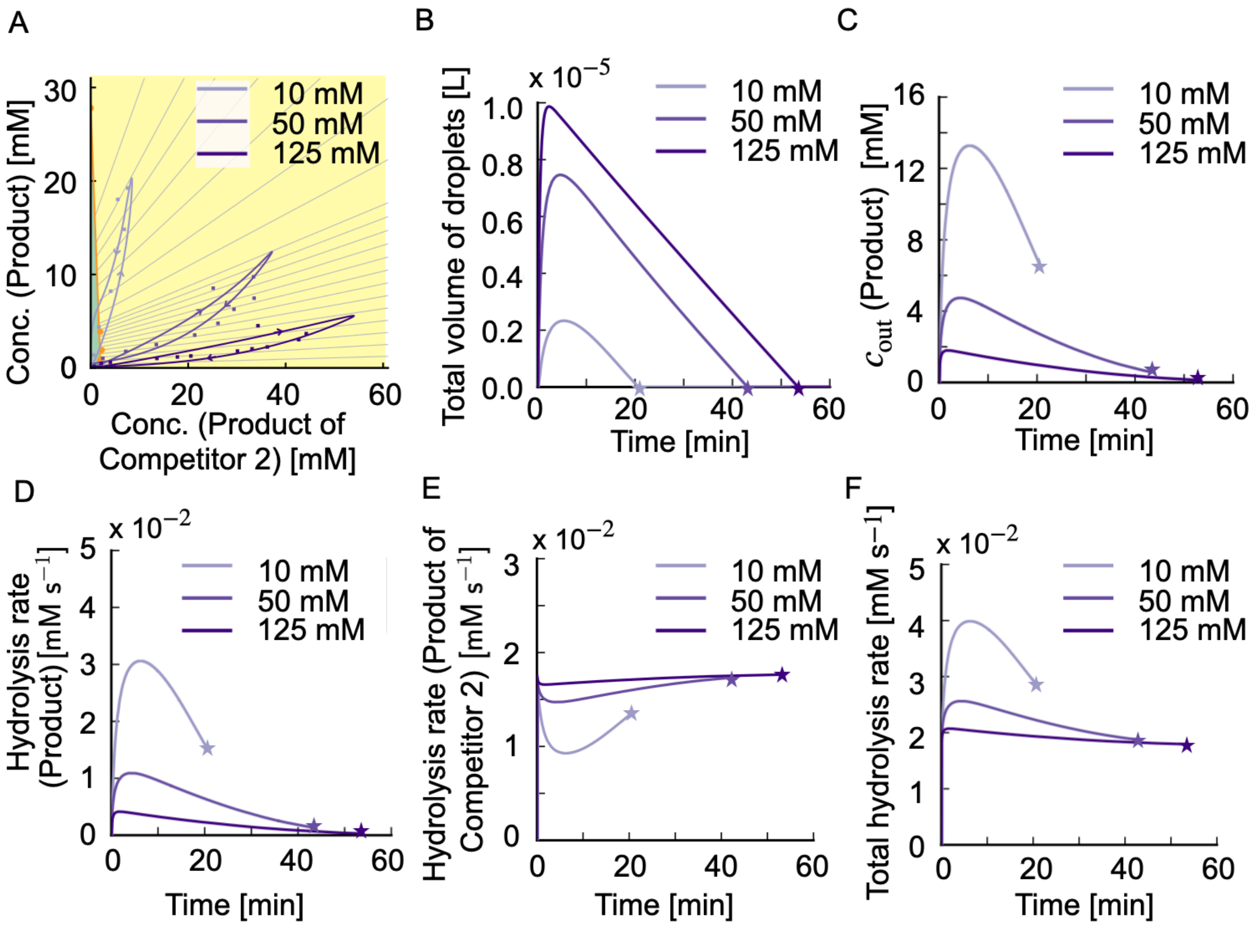

Figure 14: Total droplet volume increases and total hydrolysis rate decreases in the dilute phase allowing longer survival of the parasite. A) The kinetic orbits corresponding to different concentrations of competitor 2. The kinetic orbit crosses fewer tie lines at maximal competitor 2 concentration (125 mM), suggesting the solubility change is not drastic and allows nearly zeroth order decay for both anhydrides. Markers represent HPLC data; solid lines represent data calculated using the theoretical kinetic model. B) The total droplet volume increases with increasing the competitor 2 concentration and the product of competitor 2 starts acting like a host to protect the parasite inside the droplets from hydrolysis. C) The equilibrium concentration of the product in the aqueous phase, c_{out} , decreases with increasing competitor 2 concentration. D) The hydrolysis rate of the product in the dilute phase decreases with increasing competitor 2 concentration. E) The hydrolysis rate of the product of competitor 2 in the aqueous phase sets the offset of the total hydrolysis rate, and it increases with increasing competitor 2 concentration. F) The total hydrolysis rate of both anhydrides outside the droplets. The stars in B, C, D, E and F denote the time-point of dissolution of droplets.

7. Co-phase separation with periodic fuelling

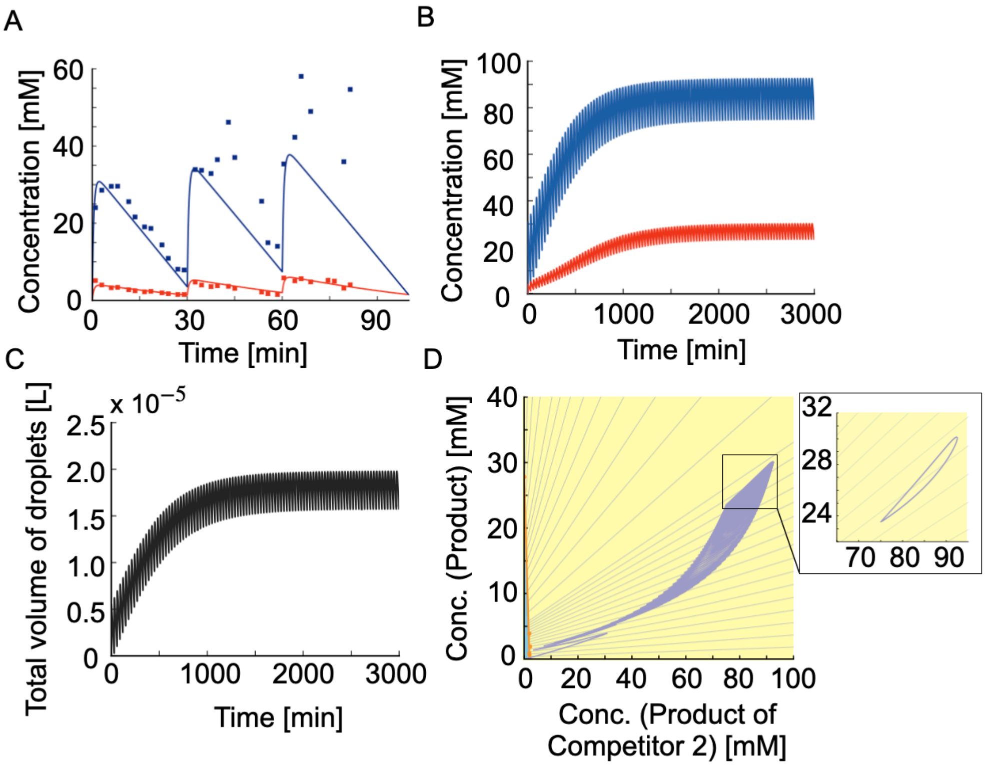

Figure 15: Survival and consequent enrichment of both the product of competitor 2 (host) and the product (parasite) due to co-phase separation. A) The time traces of both anhydrides (host and parasite with initial concentrations 100 mM and 50 mM, respectively), for periodic fuelling at the rate of 60 mM every 30 minutes. Markers represent HPLC data; solid lines represent data calculated using the theoretical kinetic model. Disagreement between the experimental data and the data calculated from the kinetic model for the host is more prominent after first cycle of fuelling due to formation of multiple droplets that settled at the bottom of the HPLC screw cap vial. B) The time traces of the host and the parasite under the experimental fuelling conditions to highlight the long time behaviour using the kinetic model. We observe the emergence of a non-equilibrium steady state for both the host and the parasite. C) The total volume of droplets also achieves a non-equilibrium steady state. D) The time trace representation in the phase diagram shows the oscillatory behaviour and approach to a non-equilibrium steady state. The inset depicts the limit cycle in the phase diagram achieved due to emergence of non-equilibrium steady states for both host and parasite (in the inset: last 3 cycles).

8. Buffer capacity in presence of periodic fuelling

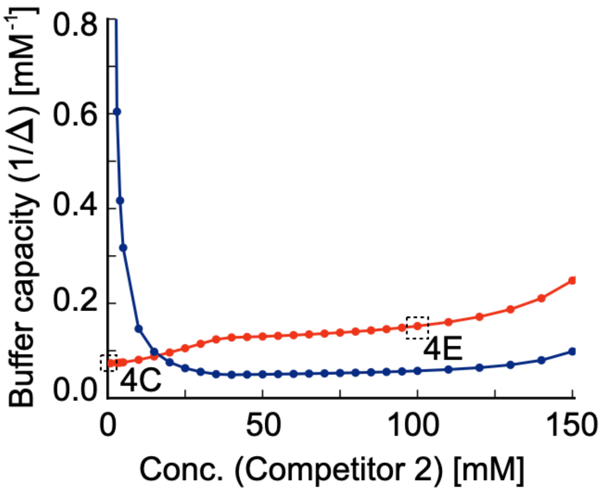

Figure 16: The buffer capacity against fuel oscillations increases for the parasite with increasing host concentration. The buffer capacity defined as inverse of the deviation in concentration, i.e., (Δ^{-1}) increases for the parasite with the increasing competitor 2 (host) concentration. The propensity to co-phase separate increases in the system with increasing the host concentration which allows for more protection of the parasite and thus less degradation. It leads to smaller deviations around mean psuedo-steady state concentration. The host's buffer capacity decreases, making it more susceptible to fluctuations. The blue and red solid line represent buffer capacity of host and parasite respectively. Initially the buffer capacity of the host is high due to lower mean concentration of the host and hence less deviation. As the mean concentration increases, the deviation around it also increases, thus reducing its buffer capacity. The opposite trend occurs for the parasite, upto 40 mM, following which the mean concentration of the product also increases due to the protection from the host droplets. The highlighted values of buffer capacity are corresponding to the data shown in the main text Figs. 4C and 4E, respectively.

9. Effects of activation rate constants on host-parasite identity

We have shown that the kinetic orbit of the average product concentrations in the phase diagram determines the lifetimes of the products and the composition inside droplets. The shape of the orbit is also affected by the rate constants. To illustrate this aspect we considered, precursor concentration of 50 mM and competitor 2 concentration of 100 mM fuelled with 100 mM EDC, as the experimental reference and swapped the rate constants related to the activation reaction pathway (Fig. 17).

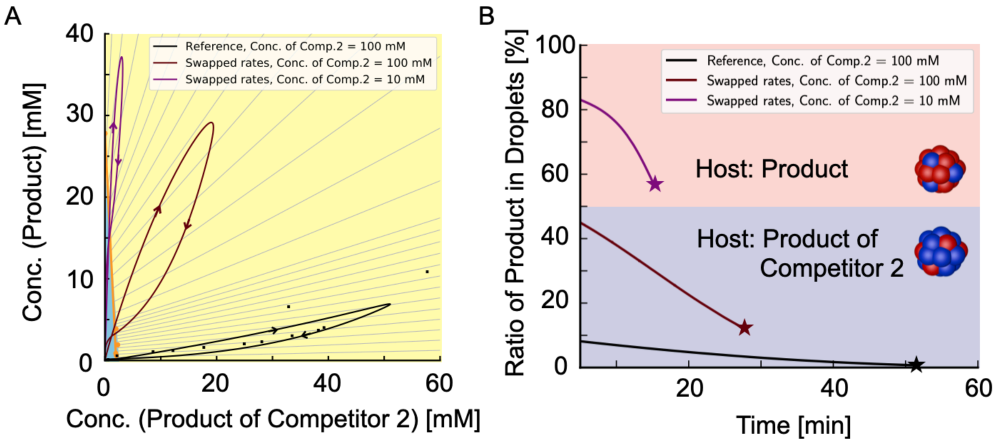

Figure 17: Host and parasite identity depends on the solubilities of the components and initial precursor concentrations. A) Kinetic orbits in the phase diagram corresponding to three different parameter sets in which we swapped the rate constants of the fuel-driven activation pathway (i.e., all rate constants except for the deactivation rate constants) and considered different concentrations of competitor 2 at a fixed EDC concentration of 100 mM and precursor concentration of 50 mM. Markers represent HPLC data; solid lines represent data calculated using the theoretical kinetic model. B) The ratio of product of precursor in droplets over time shows that the identity of host and parasite can change with swapped rates. Due to the higher solubility, the product is typically the parasite, except when it is at excess. In this case, the product starts as a host and transits to a parasite, as long as droplets do not dissolve beforehand. The star markers denote the time-point of droplet dissolution.
